# Landscape and selection of vaccine epitopes in SARS-CoV-2

**DOI:** 10.1186/s13073-021-00910-1

**Published:** 2021-06-14

**Authors:** Christof C. Smith, Kelly S. Olsen, Kaylee M. Gentry, Maria Sambade, Wolfgang Beck, Jason Garness, Sarah Entwistle, Caryn Willis, Steven Vensko, Allison Woods, Misha Fini, Brandon Carpenter, Eric Routh, Julia Kodysh, Timothy O’Donnell, Carsten Haber, Kirsten Heiss, Volker Stadler, Erik Garrison, Adam M. Sandor, Jenny P. Y. Ting, Jared Weiss, Krzysztof Krajewski, Oliver C. Grant, Robert J. Woods, Mark Heise, Benjamin G. Vincent, Alex Rubinsteyn

**Affiliations:** 1 Department of Microbiology and Immunology, UNC School of Medicine, Chapel Hill, NC USA; 2 Lineberger Comprehensive Cancer Center, University of North Carolina at Chapel Hill, CB# 7295, Chapel Hill, NC 27599-7295 USA; 3 Department of Genetics and Genomic Sciences, Icahn School of Medicine at Mount Sinai, New York, NY USA; 4 PEPperPRINT GmbH, Heidelberg, Germany; 5 Genomics Institute, University of California, Santa Cruz, CA USA; 6 Department of Genetics, UNC School of Medicine, Chapel Hill, NC USA; 7 Institute for Inflammatory Diseases, University of North Carolina at Chapel Hill, Chapel Hill, NC USA; 8 Center for Translational Immunology, University of North Carolina at Chapel Hill, Chapel Hill, NC USA; 9 Division of Medical Oncology, Department of Medicine, UNC School of Medicine, Chapel Hill, NC USA; 10 Department of Biochemistry and Biophysics, UNC School of Medicine, Chapel Hill, NC USA; 11 Complex Carbohydrate Research Center, University of Georgia, Athens, GA USA; 12 Computational Medicine Program, UNC School of Medicine, Chapel Hill, NC USA; 13 Curriculum in Bioinformatics and Computational Biology, UNC School of Medicine, Chapel Hill, NC USA; 14 Division of Hematology, Department of Medicine, UNC School of Medicine, Chapel Hill, NC USA

**Keywords:** SARS-CoV-2, COVID-19, vaccine, T cell, B cell

## Abstract

**Background:**

Early in the pandemic, we designed a SARS-CoV-2 peptide vaccine containing epitope regions optimized for concurrent B cell, CD4^+^ T cell, and CD8^+^ T cell stimulation. The rationale for this design was to drive both humoral and cellular immunity with high specificity while avoiding undesired effects such as antibody-dependent enhancement (ADE).

**Methods:**

We explored the set of computationally predicted SARS-CoV-2 HLA-I and HLA-II ligands, examining protein source, concurrent human/murine coverage, and population coverage. Beyond MHC affinity, T cell vaccine candidates were further refined by predicted immunogenicity, sequence conservation, source protein abundance, and coverage of high frequency HLA alleles. B cell epitope regions were chosen from linear epitope mapping studies of convalescent patient serum, followed by filtering for surface accessibility, sequence conservation, spatial localization near functional domains of the spike glycoprotein, and avoidance of glycosylation sites.

**Results:**

From 58 initial candidates, three B cell epitope regions were identified. From 3730 (MHC-I) and 5045 (MHC-II) candidate ligands, 292 CD8^+^ and 284 CD4^+^ T cell epitopes were identified. By combining these B cell and T cell analyses, as well as a manufacturability heuristic, we proposed a set of 22 SARS-CoV-2 vaccine peptides for use in subsequent murine studies. We curated a dataset of ~ 1000 observed T cell epitopes from convalescent COVID-19 patients across eight studies, showing 8/15 recurrent epitope regions to overlap with at least one of our candidate peptides. Of the 22 candidate vaccine peptides, 16 (n = 10 T cell epitope optimized; n = 6 B cell epitope optimized) were manually selected to decrease their degree of sequence overlap and then synthesized. The immunogenicity of the synthesized vaccine peptides was validated using ELISpot and ELISA following murine vaccination. Strong T cell responses were observed in 7/10 T cell epitope optimized peptides following vaccination. Humoral responses were deficient, likely due to the unrestricted conformational space inhabited by linear vaccine peptides.

**Conclusions:**

Overall, we find our selection process and vaccine formulation to be appropriate for identifying T cell epitopes and eliciting T cell responses against those epitopes. Further studies are needed to optimize prediction and induction of B cell responses, as well as study the protective capacity of predicted T and B cell epitopes.

**Supplementary Information:**

The online version contains supplementary material available at 10.1186/s13073-021-00910-1.

## Background

SARS-CoV-2 vaccines have largely focused on generation of B cell responses to trigger production of neutralizing antibodies [1–3]. SARS-CoV-2 enters cells through interaction of the viral receptor binding domain (RBD) with angiotensin-converting enzyme 2 (ACE2) receptors, found on the surface of human nasopharyngeal, lung, and gut mucosa [4]. Neutralizing antibodies targeting the RBD and other functional domains of the SARS-CoV-2 spike protein are a major route for achieving immunity and vaccine efficacy [5–10]. When work on this study began in March 2020, little was known about the relative contribution of different adaptive immune compartments to immunity against SARS-CoV-2. Broadly, it was understood that CD4^+^ and CD8^+^ T cells have roles in the antiviral immune response, including against SARS-CoV-1 [11–13]. Prior studies in SARS-CoV-1 have demonstrated T cell responses against viral epitopes, with strong T cell responses correlated with generation of higher neutralizing antibody titers [13]. Unlike antibody epitopes, T cell epitopes need not be limited to accessible regions of surface proteins. In SARS-CoV-1, concurrent CD4^+^ and CD8^+^ activation and central memory T cell generation were induced in exposed patients, with increased Th2 cytokine polarization observed in patients with fatal disease [13]; conversely, Th1 response has been associated with less severe disease in SARS-CoV-2 [14]. Additionally, Type 1 and Type 2 immunity are not strictly synonymous with cell-mediated and humoral immunity, respectively, with Th1 polarization capable of inducing moderate antibody production [15]. Because of these considerations, most groups developing vaccines for SARS-CoV-2 have focused on promoting Th1 response due to safety concerns and demonstrated efficacy of Th1 response [16]. To this end, we deduced that vaccines targeting humoral (B cells) and cytotoxic arms (CD8^+^ T cells) with concurrent helper signalling (CD4^+^ T cells), delivered with adjuvants promoting Th1 polarization, may provide optimal immunity against SARS-CoV-2.

In the intervening year, many vaccine strategies for SARS-CoV-2 have demonstrated efficacy in clinical trials, including mRNA encoding of the spike glycoprotein, recombinant spike protein, adenovirus vector expressing the surface glycoprotein, as well as delivery of whole inactivated virus [2, 3, 17–25]. These strategies have proven successful at eliciting neutralizing antibody responses against conformational epitopes [26] and offer impressive protection from both infection and disease [22, 23, 27, 28]. More recently, however, concern has emerged regarding the rapid evolution [29, 30] of the virus with concomitant decrease or loss of neutralization from some novel variants [31–33]. Currently circulating variants, however, do not appear to abrogate T cell reactivity [34] and there is hope that vaccine induced T cell responses provide a second line of defense against viral infection [35, 36]. Whether future variants would also be recognized by T cell evolutionary pressure to escape T cell responses is unclear. Multi-epitope peptide vaccination is an alternative approach which targets smaller antigenic fragments of viral proteins. Peptide vaccines have historically been most successful at eliciting T cell responses [37–40] and, in certain pathogens, they have also been able to elicit neutralizing antibodies against linear epitopes [41–44]. Peptide vaccines may have a complementary role relative to existing SARS-CoV-2 vaccines due to their history of safe administration [45–48], rapid development [49, 50], and precise selection of antigenic content. A peptide vaccine can easily exclude polymorphic antigenic regions or be updated to include antigenic fragments from newly emerging variants.

**Fig. 1:**
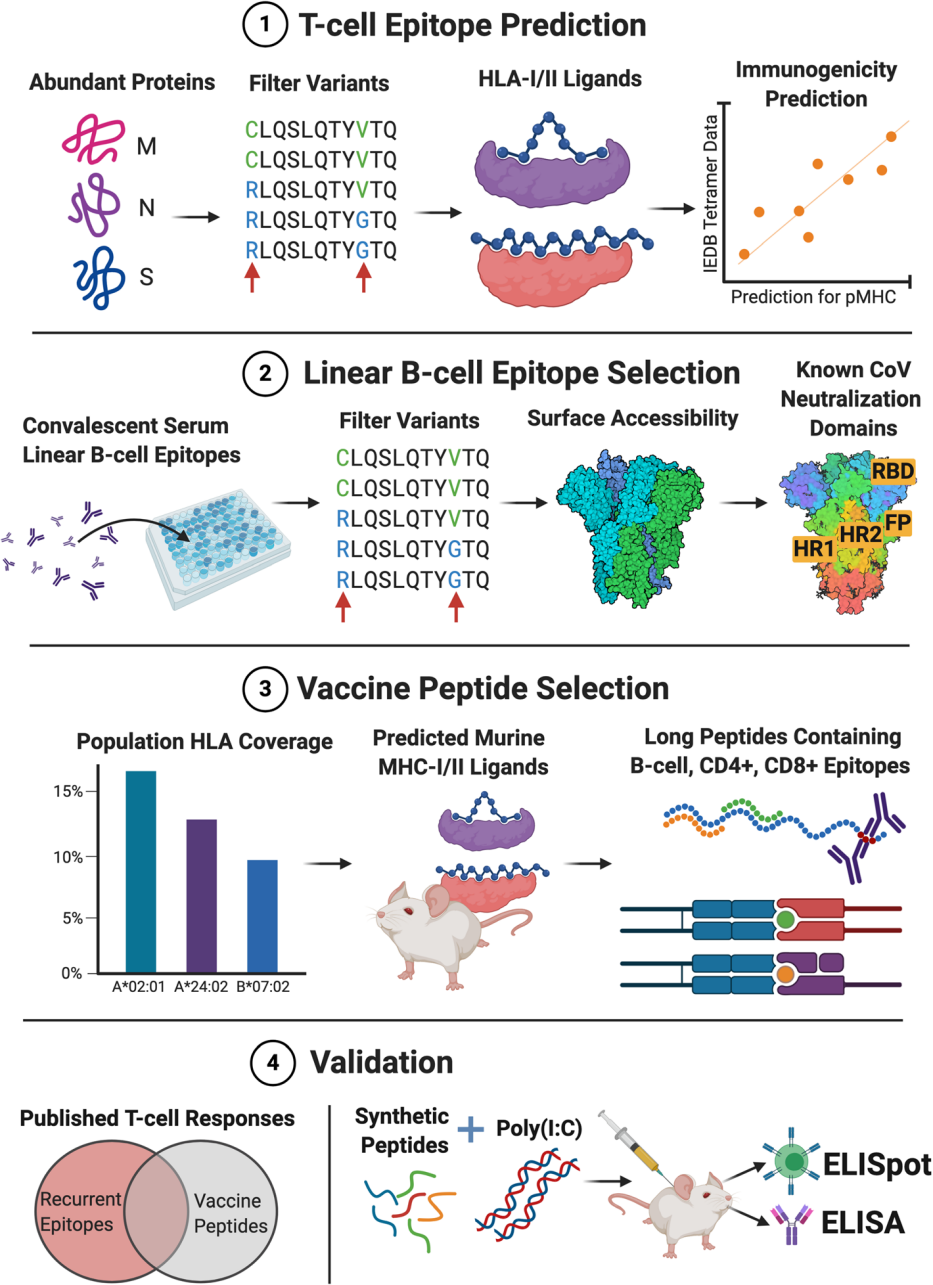
Visual summary of T and B cell epitope vaccine prediction and validation. (1) We explored the set of computationally predicted SARS-CoV-2 HLA-I and HLA-II ligands, examining source protein abundance, sequence conservation, coverage of high frequency HLA alleles, and predicted immunogenicity. (2) B cell epitope regions were chosen from linear epitope mapping studies of convalescent patient serum, followed by filtering for sequence conservation, surface accessibility, spatial localization near functional domains of the spike glycoprotein, and avoidance of glycosylation sites. (3) Vaccine selection of 27mers peptides was performed by optimizing population HLA coverage of T cell epitopes, evaluating human/murine MHC ligand co-coverage, as well as examining peptides with optimal coverage of B cell, CD4^+^, and CD8^+^ epitopes. (4) Lastly, validation was performed through comparison against a curated dataset of ~ 1000 observed T cell epitopes from convalescent COVID-19 patients across eight studies, as well as murine ELISA/ELISpot studies using animals vaccinated with synthetic 27mer peptides with human/murine epitope co-coverage

**Fig. 2:**
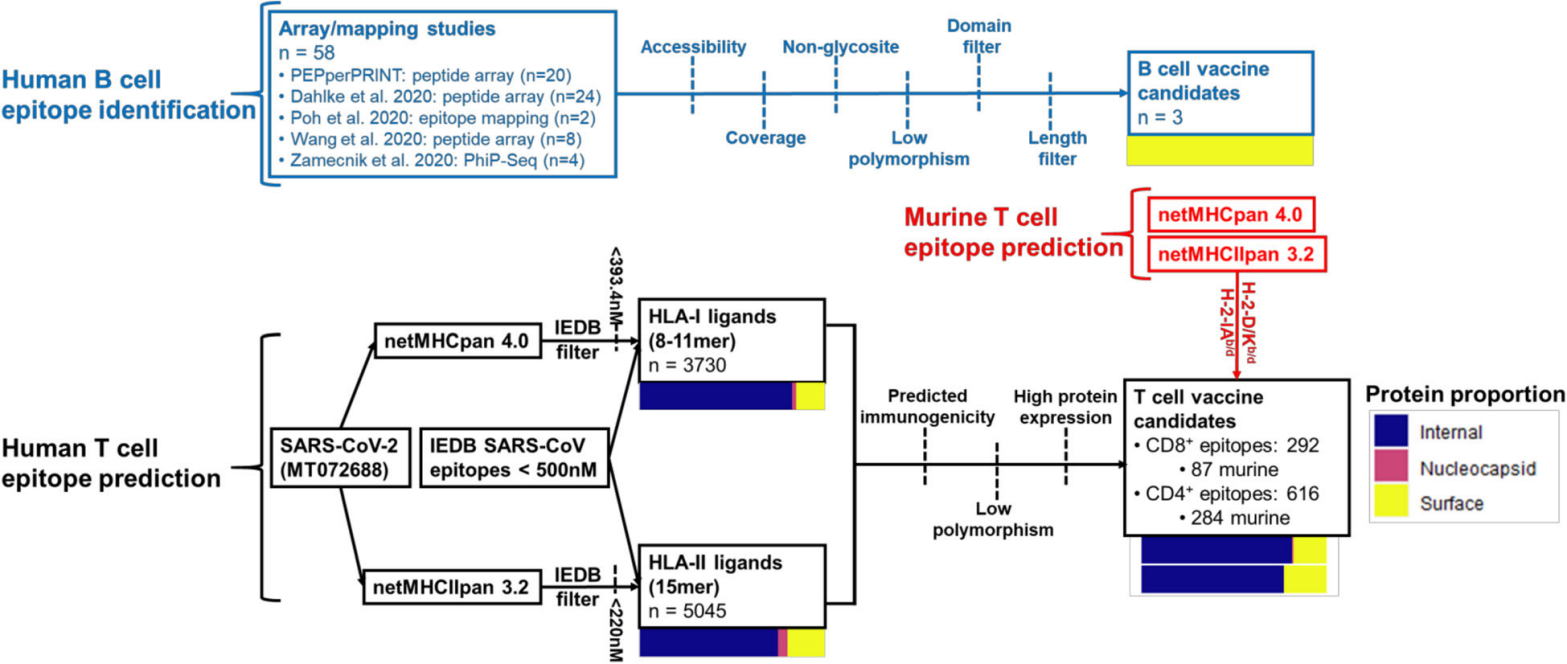
Summary of B cell and CD4^+^/CD8^+^ epitope prediction workflows. Pathways are colored by B cell (blue), human T cell (black), and murine T cell (red) epitope prediction workflows. Color bars represent proportions of epitopes derived from internal proteins (ORF), nucleocapsid phosphoprotein, and surface-exposed proteins (spike, membrane, envelope)

We report here a design methodology for selecting SARS-CoV-2 vaccine peptides which combines linear B cell epitopes with both CD4^+^ and CD8^+^ T cell epitopes, as well as an evaluation of our strategy based on a murine vaccination study and a comparison with a curated dataset of published SARS-CoV-2 T cell epitopes (Fig. 1). We start with a survey of the T and B cell epitope space of SARS-CoV-2 (Fig. 2). Predicted T cell epitopes were derived from in silico predictions filtered on binding affinity and immunogenicity models generated from epitopes deposited in the Immune Epitope Database (IEDB) [51], population diversity, and source protein abundance in order to select peptides that bind common HLA alleles and are likely to generate robust CD8^+^ and CD4^+^ T cell activity. B cell epitope candidates were curated from linear epitope mapping studies and further filtered by accessibility, glycosylation, polymorphism, and adjacency to functional domains to identify peptides most likely to generate robust antibody responses. Given the utility of murine-adapted SARS-CoV-2 models for evaluating vaccine candidates [7, 52–54], we also identified peptides derived from viral proteins predicted to bind murine MHC coded for by H2-D^b/d^, H2-K^b/d^, and H2-IA^b/d^ haplotypes. We then selected 22 longer sequence regions for use as vaccine antigens. These vaccine peptides each span multiple predicted CD4^+^/CD8^+^ T cell and linear B cell epitopes, along with predicted murine MHC-I/II ligands. We compared this vaccine peptide selection process with a curated dataset of eight studies mapping SARS-CoV-2 T cell epitopes from COVID-19 patients and found that many of the recurrent epitope regions were captured by our vaccine peptides. We also evaluated 16 of the 22 vaccine peptides in a murine vaccination experiment and found that the same subset of the peptides elicited T cell responses in combination with two different adjuvants.

## Methods

### Antibody epitope curation

Linear B cell epitopes on the SARS-CoV-2 surface glycoprotein were curated from five published studies [55–59]. Four of these studies screened polyclonal sera of convalescent COVID-19 patients using either peptide arrays [55, 56, 59] or phage immunoprecipitation sequencing (PhIP-Seq) [57]. One study characterized the epitopes of monoclonal neutralizing antibodies [59]. Results from Schwarz et al*.* included sera from six SARS-CoV-2-naive patient sera and nine SARS-CoV-2-infected patient sera using PEPperCHIP® SARS-CoV-2 Proteome Microarrays [59]. The peptides included in these proteome-wide epitope mapping analyses were limited to those which demonstrated either IgG or IgA fluorescence intensity > 1000 U in at least two infected patient samples and in none of the naive patient samples. In addition, two peptides were also included (QGQTVTKKSAAEASK, QTVTKKSAAEASKKP) which demonstrated IgG fluorescence intensity > 1000 U in only one naive patient sample each, but in four and five infected patient samples, respectively.

### HLA ligand prediction

The SARS-CoV-2 protein sequence FASTA was retrieved from the NCBI reference database (https://www.ncbi.nlm.nih.gov/nuccore/MT072688) [60]. Haplotypes included in this analysis were derived from those with > 5% expression within the United States populations based on the National Marrow Donor Program’s HaploStats tool [61]:

- HLA-A: A*11:01, A*02:01, A*01:01, A*03:01, A*24:02
- HLA-B: B*44:03, B*07:02, B*08:01, B*44:02, B*44:03, B*35:01
- HLA-C: C*03:04, C*04:01, C*05:01, C*06:02,C*07:01, C*07:02
- HLA-DR: DRB1*01:01, DRB1*03:01, DRB1*04:01, DRB1*07:01, DRB1*11:01, DRB1*13:01, DRB1*15:01
  Additionally, HLA-DQ alpha/beta pairs were chosen based on prevalence in previous studies [62]:
- HLA-DQ: DQA1*01:02/DQB1*06:02, DQA1*05:01/DQB1*02:01, DQA1*02:01/DQB1*02:02, DQA1*05:05/DQB1*03:01, DQA1*01:01/DQB1*05:01, DQA1*03:01/DQB1*03:02, DQA1*03:03/DQB1*03:01, DQA1*01:03/DQB1*06:03
  For HLA-I, 8-11mer epitopes were predicted using netMHCpan 4.0 [63] and MHCflurry 1.6.0 [64]. For HLA-II calling, 15mers were predicted using NetMHCIIpan 3.2 [65] and NetMHCIIpan 4.0 [66]. For optimization of epitope predictions, individual features from each HLA-I and HLA-II prediction tool was compared against IEDB binding affinities using Spearman correlation (Additional file 1: Fig. S1). Cutpoints for the best performing HLA-I and HLA-II feature were set using 90% specificity of predicting for peptides with < 500 nM binding affinity in the IEDB set, using predicted binding affinity values from netMHCpan 4.0 (HLA-I) and netMHCIIpan 3.2 (HLA-II). The proportion of the total U.S. population containing at least one haplotype capable of binding each peptide was calculated assuming no genetic linkage:
  $$ 1-\prod \limits_i{\left(1-{f}_i\right)}^2 $$

### Immunogenicity modeling

IEDB HLA-I and HLA-II viral tetramer data were used to generate a generalized linear model (GLM; family = binary) with tetramer-positivity as a binary outcome [51]. Independent variables for HLA-I included NetMHCpan 4.0 binding affinity and elution score, MHCflurry binding affinity, presentation score, processing score, and percentage of aromatic (F, Y, W), acidic (D, E), basic (K, R H), small (A, G, S, T, P), cyclic (P), and thiol (C, M) amino acid residues. Independent variables for HLA-II included NetMHCIIpan 4.0 binding affinity and elution scores, and percentage of aromatic, acidic, basic, small, cyclic, and thiol amino acid residues. All independent variables were normalized to 0–1 to keep coefficients comparable (binding affinities divided by 50,000). GLM model performance was derived using 5-fold cross-validation, balancing for HLA alleles. The final HLA-I and HLA-II models were generated using each full IEDB set, then applied to SARS-CoV-2 predicted HLA ligands to derive a GLM score. For immunogenicity filtering, predicted epitopes above the median GLM score were kept.

### B cell epitope selection

Accessibility of contiguous regions of the spike protein was approximated with the following heuristic: mean accessibility of 35%, minimum accessibility of 15%, requiring at least one residue to have accessibility greater than 50%, and the ends of a region to have at least 25% accessibility. Adjacency to a functional region was defined as within 15aa of either side of FP, HR1, and HR2, and within 50aa of the RBD. A broader window was used for the receptor binding domain due to the known presence of neutralizing antibody epitopes in S1 of SARS-CoV-1 outside of the RBD [67].

### Published T cell epitope data curation

T cell epitopes from eight studies of immune responses from convalescent COVID-19 patients [68–75] were manually curated into a spreadsheet with 973 entries (Table S9). Other studies were excluded which focused on murine immune responses and/or immunity from vaccination. To aggregate epitope regions of varying granularities, the viral proteome was split into 40aa bins, overlapping by 20aa. A bin was considered to contain an epitope region if they overlapped by at least 8aa. Similarly, each vaccine peptide counted as overlapping a bin if their overlap was at least 8aa. Overlapping bins were mutually exclusive, and only the bin with the highest number responding patients was retained. Bin boundaries were then clipped to the minimum and maximum boundaries of any epitope region contained within it.

### Vaccine peptide manufacturability

Based on previous experiences with peptide synthesis failures and consultation with the UNC High-Throughput Peptide Synthesis and Array Facility, we devised a scoring rubric for solid-phase peptide synthesis difficulty (Additional file 1: Fig. S8A). This rubric includes features related to the stability of the synthesized peptide product as well as sequence features which increase the difficulty of peptide elongation and/or purification. For example, hydrophobic peptides are challenging to solubilize, whereas hydrophobic regions within peptides are challenging to elongate during synthesis due to strong conformational properties. In our scoring rubric, hydrophobicity of peptide sequences is calculated using the mean GRAVY score [76], which is computed both for the entire peptide as well as the max for all local windows of lengths between 5mer and 8mer. Local hydrophobicity scores are penalized proportional to how much they exceed 2.5 whereas whole peptide hydrophobicity is penalized to the degree that it exceeds 2. These values were determined based on unpublished data relating to which peptides had failed for reasons related to hydrophobicity during the PGV001 neoantigen vaccine trial [77]. Another category of difficulties relates to the instability of certain pairs of adjacent amino acids. The extremely unstable dipeptides are DG and NG, whereas the less penalized but still problematic dipeptides are DS, DN, DD, NN, ND, NS, and NP. Furthermore, certain terminal residues inhibit the initiation of synthesis or formation of undesired residues such as pyroglutamate. Difficult N terminal residues are Q, E, C, and N, whereas difficult C terminal residues are P, C, and H. Lastly, the inclusion of multiple thiol residues can be challenging due to formation of long-range disulfide bonds. Our heuristic penalizes both the total number of thiols (C and M residues), as well as a penalty for excessive cysteines which is only applied when the number of C residues exceeds 1. Many of similar features are enumerated in commercial peptide design guides, such as ones published by Biomatik [78] and SB peptide [79] or in standard texts on solid-phase synthesis [80]. The particular weights given to different peptide features are determined purely from experience and intuition and are presented without claims of accuracy or optimality.

### SARS-CoV-2 entropy calculations

In total, 7881 SARS-CoV-2 genome sequences were downloaded from GISAID (https://www.gisaid.org/) [81]. A preprocessing step removed 127 sequences that were shorter than 25,000 bases. The sequences were split into 79 smaller files and aligned using Augur [82] (which relies on the MAFFT [83] aligner) with NCBI entry MT072688.1 [84] as the reference genome. The reference genome was downloaded from NCBI GenBank [85]. The 79 resulting alignment files were concatenated into a single alignment file with the duplicate reference genome alignments removed. The multiple sequence alignment was translated to protein space using the R packages seqinr [86] and msa [87]. Entropy for each position was calculated using the following formula, where *n* is the number of possible outcomes (i.e., total unique identifiable amino acid residues at each location) and *p*_*i*_ is the probability of each outcome (i.e., probability of each possible amino acid residues at each location):

$$ -\sum \limits_{i=1}^n{p}_i\cdot \mathit{\log}\left({p}_i\right) $$

### Mouse vaccination

All mouse work was performed according to IACUC guidelines under UNC IACUC protocol ID 20-121.0. Vaccine studies were performed using BALB/c mice with free access to food and water. Mice were ordered from Jackson Laboratories and vaccinated at 8 weeks of age. Equal numbers of male and female mice were used per group, vaccinated with poly(I:C) (Sigma-Aldrich cat. #P1530) either alone or in combination with 16 synthesized vaccine peptides. In total, 26 μg total peptide was utilized per vaccination (divided equimass per peptide). Then, 75 μg of polyI:C was utilized per vaccination, with n = 6 mice per experimental group and n = 3 mice per polyI:C-only control group. Mice were vaccinated on days 1 and 7, cheek bleeds obtained on days 7 and 14, and sacrificed with cardiac bleeds performed on day 21.

### S Protein ELISA

Serum obtained from cardiac bleeds on day 21 was utilized for ELISA testing for antibody response to SARS-CoV-2 spike (S) protein. Nunc Maxisorp plates (Thermo Fisher Scientific) were coated with S protein (generously provided by Ting Lab at UNC), or BSA as a negative control and incubated overnight. Plates were blocked with 10% FBS in PBS, washed, and serum plated in duplicate wells with serial dilutions. 6x His Tagged monoclonal antibody (Thermo Fisher Scientific) was also plated as an experimental control. Goat anti-mouse IgG HRP (Thermo Fisher Scientific) was added to washed plates as a secondary antibody. TMB substrate (Thermo Fisher Scientific) was added, development was stopped with TMB Stop solution (BioLegend), and plates were read at 450 nm.

### Peptide ELISA

Serum obtained from cardiac bleeds on day 21 and cheek bleeds on experimental days 7 and 14 were tested for antibody response to the predicted B cell peptide epitopes used for vaccinations via peptide ELISAs. Plates were coated with 5μg/mL of target peptide using coating reagent from the Takara Peptide Coating Kit (Takara cat. #MK100). Measles peptide was utilized as a negative control, and Flag peptide was also plated as an experimental control. Plates were blocked with a blocking buffer according to the manufacturer’s protocol. Serum was plated in duplicate wells with serial dilutions, and anti-FLAG antibody was plated in the experimental control wells. Rabbit anti-mouse IgG HRP (Abcam ab97046) was utilized as a secondary antibody. TMB substrate (Thermo Fisher Scientific cat. #34028) was added, development was stopped with TMB Stop solution (BioLegend cat. #423001), and plates were read at 450 nm.

### ELISpot

After the sacrifice of mice on experimental day 21, spleens were dissected out for ELISpot assessment of T cell activation in response to peptide and adjuvant vaccination. Spleens were mechanically dissociated using a GentleMACS Octo Dissociator (Miltenyi Biotec) and passed through a 70-μm filter. RBC lysis buffer (Gibco cat. #A1049201) was used to remove red blood cells, and cells were washed then passed through 40-μm filters. Splenocytes were counted and 250,000 splenocytes were plated per well into plates (BD Biosciences; cat. #551083) that had been coated with each of the individual 16 predicted target peptides, or PBS as negative control or PHA as experimental control. Plates were incubated for 72 h. Anti-interferon gamma detection antibody was added according to the manufacturer’s protocol, followed by enzyme conjugate Streptavidin-HRP and final substrate solution (BD Biosciences; cat. #557630). Plates were allowed to develop, washed to stop development, and allowed to dry before reading on ELISpot reader (AID Classic ERL07).

### Graphical and statistical analysis

Plots and analyses were generated using the following R packages: caret 6.0-84 [88], cowplot 0.9.4 [89], data.table 1.12.8 [90], DESeq2 1.22.2 [91], doMC 1.3.6 [92], dplyr 0.8.4 [93], forcats 0.4.0 [94], GenomicRanges 1.34.0 [95], ggallin 0.1.1 [96], ggbeeswarm 0.6.0 [97], ggnewscale 0.4.1 [98], ggplot2 3.3.0 [89], ggpubr 0.2 [99], ggrepel 0.8.1 [100], gplots 3.0.3 [101], gridExtra 2.3 [102], huxtable 4.7.1 [103], magrittr 1.5 [104], officer 0.3.10 [105], pROC 1.16.2 [106], RColorBrewer 1.1-2 [107], readxl 1.3.1 [108], scales 1.1.0 [109], seqinr 3.6-1 [86], stringr 1.4.0 [110], venneuler 1.1-0 [111], viridis 0.5.1 [112]. Figures 4C, D and 5 were generated using the following Python packages: NumPy [113], pandas [114], Matplotlib [115], and Jupyter [116].

## Results

### Landscape of MHC ligands in SARS-CoV-2

To determine the landscape of potential HLA ligands in SARS-CoV-2 (Fig. 2, black), we first identified candidate MHC ligands by performing HLA-I binding prediction using NetMHCpan 4.0 (both EL (elution ligand) and BA (binding affinity) mode) [63] and MHCflurry [64] (8–11mers), and HLA-II binding prediction using NetMHCIIpan 3.2 [65] and 4.0 [66] (15mers), using alleles with > 5% genetic frequency in the USA [61, 62] and worldwide populations [117] (full predicted sets for U.S. alleles: Table S1, S2; worldwide alleles: Table S3, S4). To assess the accuracy of these peptide/MHC binding prediction tools on viral peptides, we tested their performance on IEDB MHC affinity assay data values for viral peptides. Of the predictive models evaluated, NetMHCpan 4.0 (BA) and NetMHCIIpan 3.2 demonstrated the highest correlation of binding affinity predictions for Class I and Class II MHC, respectively (Additional file 1: Fig. S1A-B). Therefore, these two predictors were used for predicting MHC ligands. A measured peptide/MHC binding affinity of 500 nM or less is commonly used to identify MHC-binding peptides which are more likely to be T cell epitopes [118, 119]. To account for the inaccuracy inherent to prediction (as opposed to measurement) of peptide-MHC affinity, we derived slightly stricter cutoffs. In order to achieve 90% specificity in IEDB binding affinity data (validated ligand set), we use predicted binding affinity thresholds of 393.4 nM and 220.0 nM for Class I and Class II MHC, respectively (Additional file 1: Fig. 1C-D). This filter was applied to NetMHCpan 4.0 and NetMHCIIpan 3.2 SARS-CoV-2 MHC binding predictions, which removed the majority of viral protein sub-sequences (Additional file 1: Fig. 2A-B).

**Fig. 3:**
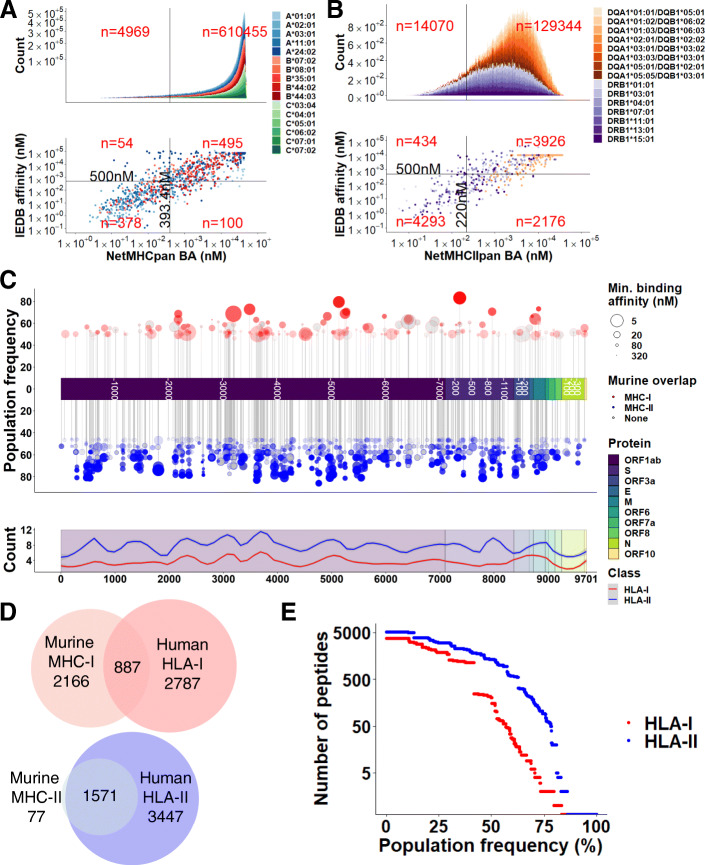
Landscape of SARS-CoV-2 MHC ligands. **A,B** Selection criteria for **A** HLA-I and **B** HLA-II SARS-CoV-2 HLA ligand candidates. Scatterplot (bottom) shows predicted (x-axis) versus IEDB (y-axis) binding affinity, with horizontal line representing 500 nM IEDB binding affinity and vertical line representing corresponding predicted binding affinity for 90% specificity in binding prediction. Histogram (top) shows all predicted SARS-CoV-2 HLA ligand candidates. Scatterplot in **B** shows subsampled points from HLA-DRB1 alleles (< 50 points per allele) to allow for increased visibility of points. **C** Landscape of predicted HLA ligands, showing HLA-I (red) and HLA-II (blue) ligands with U.S. population coverage > 50% (top), and LOESS fitted curve (span = 0.1) for HLA-I/II ligands by location along the SARS-CoV2 proteome (color tracks). The predicted binding affinity of HLA ligand peptides to murine H2-b/d alleles is represented with point shading. **D** Summary of murine/human MHC ligand overlap. **E** Distribution of population frequencies among predicted HLA-I and HLA-II ligands

After filtering by binding affinity, we observed a total of 2486 unique HLA-I ligands and 3138 unique HLA-II ligands (Fig. 3C). Predicted MHC ligands were not evenly distributed across the proteome, with local peaks and troughs observed that correlated between HLA-I and HLA-II ligands (Fig. 3C, bottom; Pearson correlation of HLA-I/II LOESS, r = 0.703, *p* < 0.001). Notably, while SARS-CoV-1 T cell epitopes previously described in the literature were primarily located in the surface glycoprotein (S) and nucleocapsid protein (N) (Table S5) [13, 120–145], we observed a paucity of predicted MHC ligands in the N protein. As murine models for SARS-CoV-2 would be a powerful tool in understanding viral immunobiology, we determined which predicted HLA ligands were also predicted to bind murine MHC alleles of the H2^b^ and H2^d^ haplotypes. NetMHCpan and NetMHCIIpan were run using the SARS-CoV-2 proteome against the H2^b^ and H2^d^ haplotypes, filtering by MHC-I ligands in the top 2nd percentile (n = 3053) and MHC-II ligands in the top 10th percentile (n = 1648). From this set, we observed an overlap of 887 peptides in MHC-I and 1571 peptides in MHC-II between murine and human sets (Fig. 3D). For the nested HLA ligand set, we observed 825 and 848 overlapping murine MHC-I and MHC-II ligands, respectively, with 846 HLA ligands containing both murine MHC-I and MHC-II coverage. The majority of HLA ligand sequences were predicted to bind to fewer than 50% of the U.S. population, particularly for HLA-I ligands (Fig. 3E). In accordance with higher population coverage distribution in HLA-II, predicted HLA-II ligands also demonstrated more binding alleles on average (mean alleles per peptide: HLA-I = 1.35, HLA-II = 2.80). Among the most common alleles were HLA-A*02:01 (n = 784), HLA-A*11:01 (n = 643), and HLA-A*03:01 (n = 383) for predicted HLA-I binding peptides and HLA-DRB1*01:01 (n = 5401), HLA-DRB1*07:01 (n = 3225), and HLA-DRB1*13:01 (n = 3022) for predicted HLA-II binding peptides.

### CD8^+^ and CD4^+^ T cell epitope prediction

**Fig. 4:**
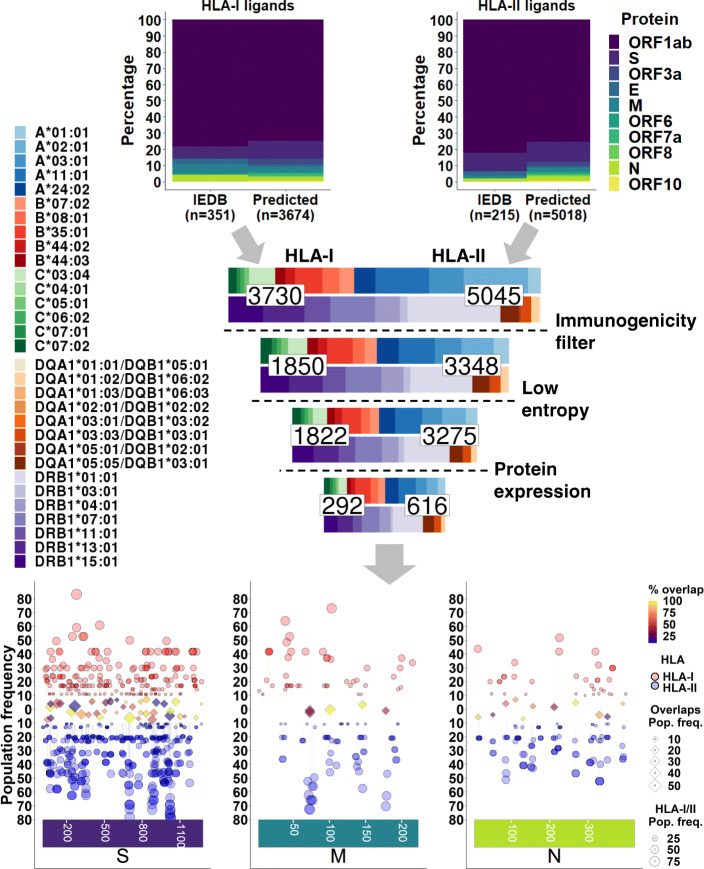
Prediction of SARS-CoV-2 T cell epitopes. (Top) Summary of predicted and IEDB-defined HLA-I (left) and HLA-II (right) SARS-CoV-2 HLA ligands, showing proportions of each derivative protein. (Middle) Funnel plot representing counts of HLA-I (left) and HLA-II (right) ligands along with proportions of HLA-I (top bar) and HLA-II (bottom bar) alleles at each filtering step. (Bottom) Summary of CD8^+^ (red, top), CD4^+^ (blue, bottom), and nested T cell epitopes (middle) after filtering criteria in S, M, and N proteins. Y-axis and size represent the U.S. population frequency of each CD8^+^ and CD4^+^ epitopes by circles. Middle track of diamonds represents overlaps between CD8^+^ and CD4^+^ epitopes, showing the overlap with greatest population frequency (size) for each region of overlap. Color of diamonds represents the proportion of overlap between CD4^+^ and CD8^+^ epitope sequences

Peptide/MHC binding is necessary but not sufficient for peptide epitopes to elicit T cell responses. We sought to identify a set of epitopes that would serve as good targets for a SARS-CoV-2 T cell vaccine. From the total pool of HLA-I, HLA-II, and nested MHC ligands, we sought to prioritize sequences which are predicted to be immunogenic from highly conserved regions of abundant viral proteins (Fig. 4, middle).

To predict the immunogenicity of MHC ligands, we fit a forward stepwise multivariable logistic regression model using peptide/HLA tetramer flow cytometry data curated from viral entries of the IEDB [51]. Tetramer data was selected for the response variable because it provides unambiguous association between a peptide and its bound MHC, and additionally tests which specific peptide/MHC is capable of eliciting a T cell response*.* Each unique peptide-MHC was encoded with features derived from epitope prediction tools as well as features relating to amino acid content (see “Immunogenicity modeling”). Epitope prediction tool features were selected to allow for consideration of predicted binding affinity alongside other tangential features such as MHC ligand elution (NetMHCpan 4.0, NetMHCIIpan 4.0) and antigen processing (MHCflurry), while amino acid content was considered due to prior studies demonstrating capacity of these features to predict for epitope immunogenicity [146, 147]. Model performance in 5-fold cross-validation demonstrated AUC values of approximately 0.7 and 0.9 for HLA-I and HLA-II, respectively, in both training and test sets Additional file 1: Fig. S2A-B). Models demonstrated cleaner separation of tetramer positive and negative groups for CD4^+^ epitopes compared to CD8^+^ (Additional file 1: Fig. S2C-D). To determine a cause for this difference in model performance, we examined predicted binding affinity scores between tetramer positive and negative epitopes, which demonstrated significantly better separation for CD4^+^ epitopes than CD8^+^ epitopes (Additional file 1: Fig. S2E-F). In accordance with this difference in binding affinity distribution, the HLA-II model showed strong association between lower binding affinity and lower predicted tetramer positivity, while the HLA-I model showed a weaker inverse association (Additional file 1: Fig. S3). Due to these binding affinity distribution differences between IEDB HLA-I and HLA-II tetramer sets, a performance-based cutoff did not allow for equal filtering of CD4^+^ and CD8^+^ epitopes. Therefore, we filtered by generalized linear model (GLM) predicted immunogenicity scores above the median in each HLA-I/II SARS-CoV-2 epitope group, which provided balanced selection while removing predicted low-immunogenicity epitopes (Additional file 1: Fig. S4).

Next, we sought to prioritize epitopes derived from regions of low sequence variation across viral strains. A position-based entropy filter was applied to all epitopes (Additional file 1: Fig. S5), keeping those with an entropy score ≤ 0.1 (~ 98% sequence identity, n = 7881) in all amino acid positions across MSA-aligned SARS-CoV-2 genomes downloaded from the GISAID database [81, 82]. High entropy was observed in the well-described spike protein D614G polymorphic site (Additional file 1: Fig. S5A, red dot). Other areas of high entropy included positions 3606, 4715, 5828, and 5865 of ORF1ab, and position 84 of ORF8 (all with entropy > 0.4). The majority of positions demonstrated > 95% sequence identity, suggesting high homology between different SARS-CoV-2 viral genomes (Additional file 1: Fig. S5B). Lastly, as the likelihood of MHC presentation is correlated with protein expression [148], we filtered epitopes to those derived from the S, M, and N proteins. These were the three highest expressed proteins based on a semi-quantitative mass spectrometry analysis of SARS-CoV-2 protein expression (PSM count/protein length;Additional file 1: Fig. S6A) [149]. This protein abundance estimation closely matched expression levels derived from SARS-CoV-2 RNA-seq data (Additional file 1: Fig. S6B) [150]. After all these filtering steps, 292 CD8^+^, 616 CD4^+^, and 423 nested T cell epitopes were predicted. We cross-filtered these epitopes against a reference peptidome of 8-11mer and 15mer peptides derived from the GRCh38 reference proteome [151] and observed no overlap. Relative proportions of HLA alleles were conserved throughout filtering (Fig. 4, middle). Full peptide sets with all filtering criteria are listed in Tables S1 (HLA-I) and S2 (HLA-II).

### B cell epitope prediction

**Fig. 5:**
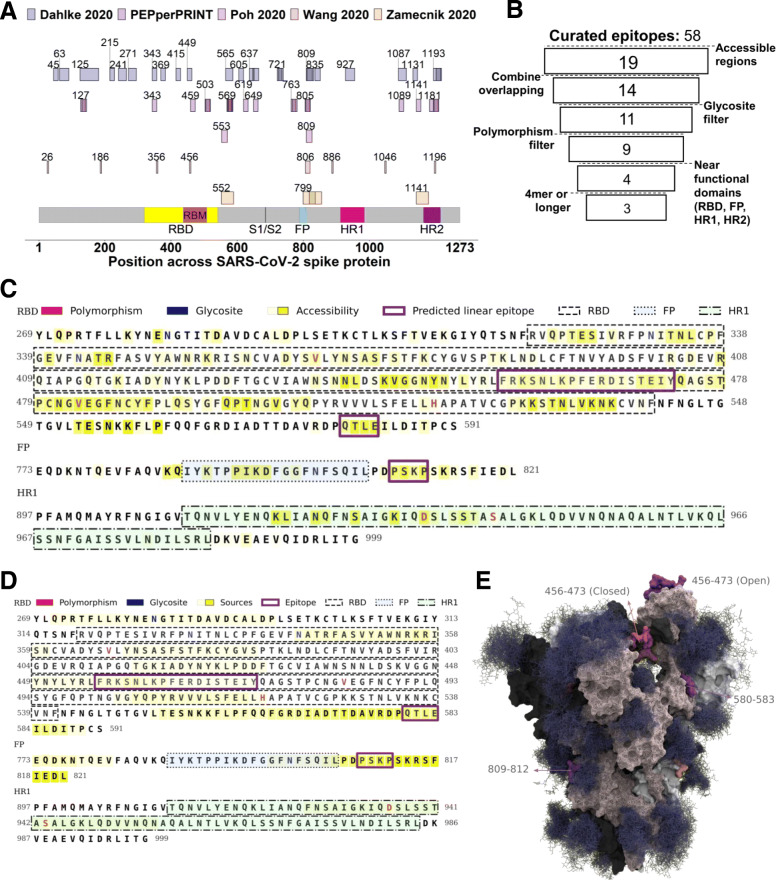
Selection of SARS-CoV-2 B cell epitope regions. **A** SARS-CoV-2 linear B cell epitopes curated from epitope mapping studies. X-axis represents amino acid position along the SARS-CoV-2 spike protein, with labeled start sites. **B** Schematic for filtering criteria of B cell epitope candidates. **C** Amino acid sequence of spike protein domains considered for B cell epitope selection, with overlay of selection features prior to filtering. Polymorphic residues are red, glycosites are blue, accessible regions highlighted in yellow. The receptor binding domain (RBD), fusion peptide (FP), and HR1 regions are outlined. HR2 excluded for lack of accessibility data. **D** Spike protein functional regions (RBD, FP, HR1) amino acid sequences, with residues colored by how many times they occur in identified epitopes. Selected accessible sub-sequences of known antibody epitopes highlighted in purple outline. **E** S protein trimer crystal structure with glycosylation, with final linear epitope regions highlighted by color

In addition to identifying SARS-CoV-2 T cell epitopes, we sought to identify a set of linear B cell epitopes on the spike protein which would serve as good targets for stimulating neutralizing antibody responses (Fig. 2). Epitope candidates were derived from four published preprint mapping/array studies [55, 56, 58, 59] including a PEPperCHIP® peptide array study [59] (for study details see “Antibody epitope curation”). Starting with an initial candidate pool of 58 linear epitopes with data to support in vivo generation in humans (Fig. 5A, Table S6), we applied a set of filtering criteria to narrow our target space (Fig. 5B):

1. Contiguous sub-sequences of the spike protein with high accessibility
2. Exclude glycosylation sites
3. Exclude regions with significant polymorphism between SARS-CoV-2 strains
4. Keep candidate epitopes within or adjacent to functional domains with evidence of antibody-mediated viral neutralization in SARS-CoV-1 (receptor binding domain, fusion peptide, heptad repeat regions)
5. Exclude any candidates shorter than four amino acids

We used SARS-CoV-2 S protein accessibility data from Grant et. al. [152], which calculates accessibility from molecular dynamics simulations of a spike protein structure with several different glycosylation patterns. Unfortunately, this accessibility data lacks HR2, causing that domain to be left out from subsequent analyses. After filtering for contiguously accessible regions, there were 19 remaining under consideration. Since many epitopes occur in multiple sources, we combined overlapping epitope candidates into 14 unique sequences. After filtering out epitopes containing glycosites, which may alter antibody binding characteristics [153, 154], 11 non-glycosylated regions remained. Two additional regions were removed because they contained polymorphic sites, defined by mutation frequency > 0.1% from GISAID SARS-CoV-2 viral sequences. Of the remaining 9 regions, only 4 were close to functional domains which in the closely related virus SARS-CoV-1 have evidence of antibody-mediated viral neutralization: the RBD, fusion protein (FP), and heptad repeats [155–160]. This filtering resulted in four remaining regions, of which our final criteria removed one which had length less than four residues (Fig. 5B). This filtering criteria precluded the vast majority of total spike protein regions (Fig. 5C), with three predicted antibody binding regions (residue lengths 18, 4, and 4) remaining (Fig. 5D). All three epitope candidate regions were present on solvent-exposed surfaces of the S protein trimer 3D structure (Fig. 5E). It is worth noting that the largest region, residues 456-473 within the receptor binding motif (RBM) loop, is only accessible when the RBD is in the “open” conformation.

### Selection of human and murine SARS-CoV-2 vaccine peptides

**Fig. 6:**
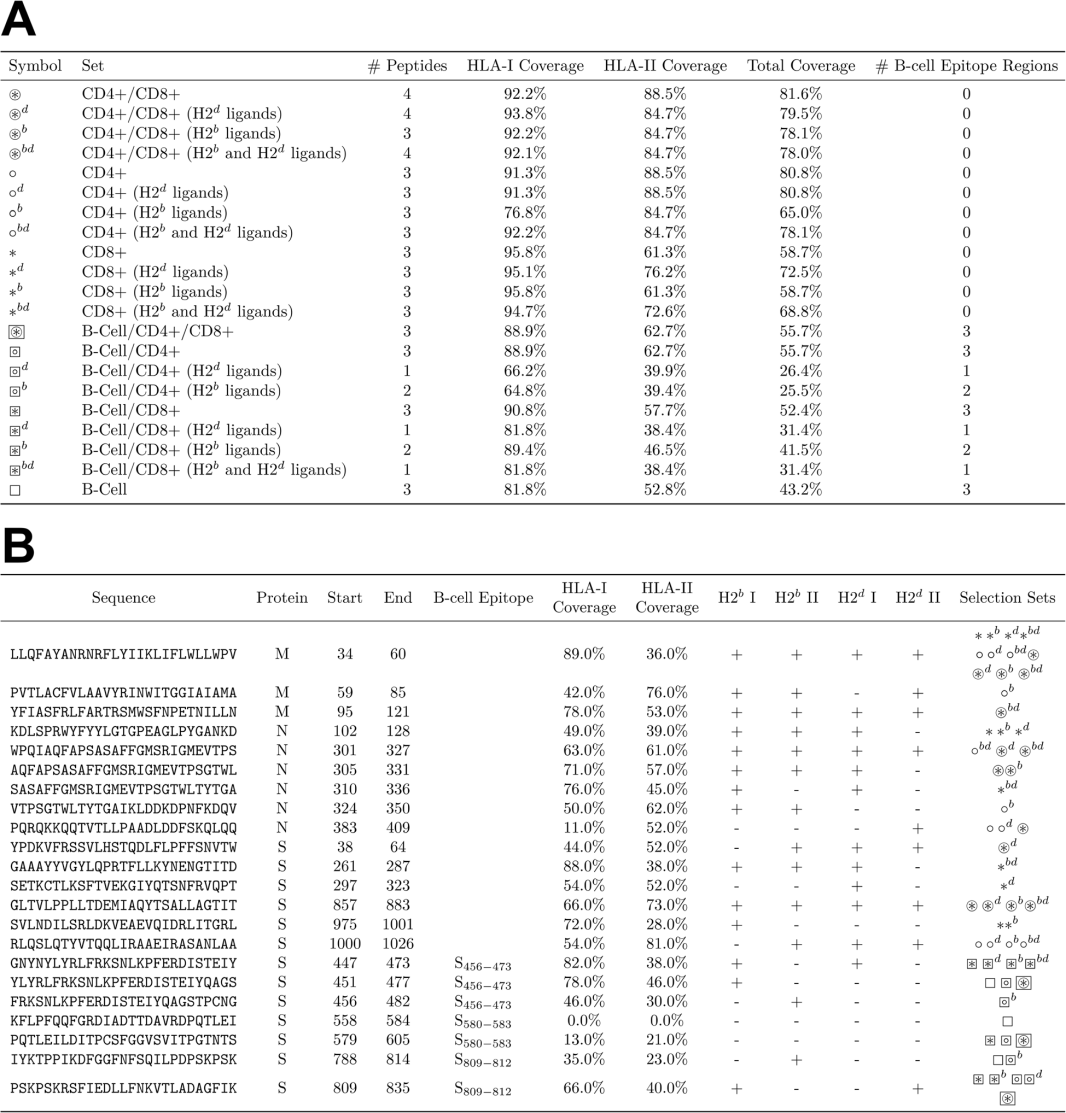
T cell and B cell vaccine candidates. **A** 27mer vaccine peptide sets selecting for best CD4^+^, CD8^+^, CD4^+^/CD8^+^, and B cell epitopes with HLA-I, HLA-II, and total U.S. population coverage. **B** Unified list of all selected 27mer vaccine peptides. Vaccine peptides containing predicted ligands for murine MHC alleles (H2-b and H2-d haplotypes) are indicated in their respective columns

With the above filters applied to predicted T and B cell epitope candidates, we derived a minimal collection of long vaccine peptides for all combinations of the following immunological criteria: CD4^+^ responses, CD8^+^ responses, coverage of predicted B cell epitopes, along with optional inclusion of predicted murine MHC ligands. A 27mer sequence for each vaccine peptide was selected to maximize U.S. population coverage of T cell epitopes within a peptide set, with or without additional coverage for murine H2^b^, H2^d^, or both haplotypes (Fig. 6A-B; Additional file 1: Fig. S7). If population coverage was identical for multiple candidates, peptides were also optimized based on a manufacturability difficulty scoring system (Additional file 1: Fig. S8). The peptide sequence length was inspired by previous work in cancer neoantigen vaccination [161–163] which has demonstrated strong CD8^+^ and CD4^+^ responses using 27mer peptides. Optimizing for CD4^+^ epitope population coverage demonstrated 88.5% population frequency encompassed by three 27mer peptides (Fig. 6B: 1, 9, and 15), while CD8^+^ epitope optimization provided 95.8% population frequency coverage by three 27mer peptides (Fig. 6B: 1, 4, and 14). CD4^+^/CD8^+^ co-optimization provided the best overall population coverage at 81.6% population frequency with four 27mer peptides (Fig. 6B: 1, 6, 9, 13). While B cell epitope optimization provided CD8^+^ coverage above 85%, CD4^+^ coverage was only 52.8%, suggesting the design of a combination B cell/CD4^+^ T cell vaccine requires use of non-spatially overlapping sequences. Overall, selection of peptides which also provided both H2^b^ and H2^d^ epitope coverage did not greatly impact population coverage, suggesting these murine-encompassing sets may allow for vaccine studies in animal models whilst preserving human relevance. Across the different selection criteria for minimal vaccine peptide sets, there was significant redundancy. Collapsing the set of vaccine peptides by unique sequences results in a final set of 22 27mer vaccine peptides (Fig. 6B). In addition to 27mer peptides, all individual T/B cell epitopes (S, M, and N: Table S7; all proteins: Table S8) as well as 15mer (Additional file 1: Fig. 9) and 21mer (Additional file 1: Fig. S10) optimized peptide sets are also available.

### Validation of T cell predictions by comparison with recurrent published T cell epitopes from COVID-19 patients

**Fig. 7:**
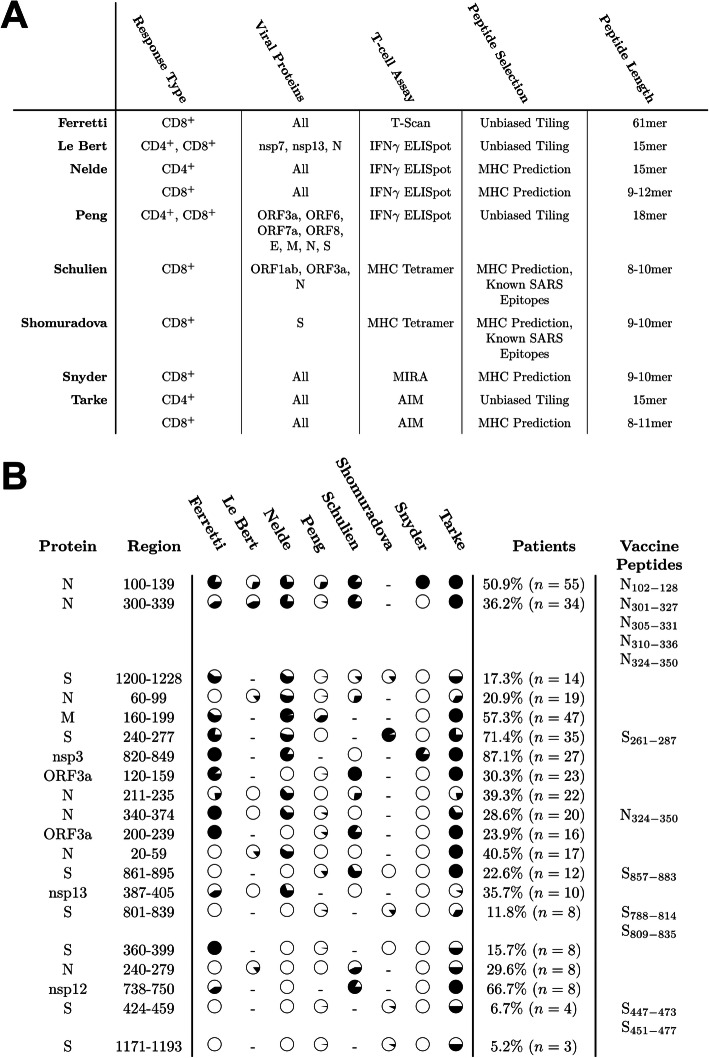
Evaluation of vaccine peptides based on published T cell responses in COVID-19 patients. **A** Overview of studies included in the T cell validation dataset. **B** All regions (up to 40aa) of the SARS-CoV-2 proteome for which at least three of the eight studies observed either a CD4+ or CD8+ T cell response. Fraction of circle fill corresponds to the largest fraction of patients with responses to any epitope in the region for a particular study. Percentage column corresponds to percent of patients with positive response to an epitope in the region as a fraction of patients evaluated. Overlapping vaccine peptides from this study are noted in the right-most column

To determine how our predictions of CD8^+^ and CD4^+^ T cell epitopes relate to actual SARS-CoV-2 T cell epitopes, we curated a dataset of published T cell epitope mapping studies (Table S9) and compared recurrent epitope regions with vaccine peptides. We focused on human studies of infection induced immunity, excluding murine and vaccine studies, as well as excluding studies which only performed TCR sequencing. We were able to curate eight diverse studies [68–74, 164] whose study characteristics are summarized in Fig. 7A. The T cell response assays included ELISpot, MHC multimers, MIRA [165], AIM [166], and T-Scan [167]. It is important to note that not all studies examined responses to the same proteins or even the same peptides within a protein. Some studies conducted exhaustive unbiased tiling over the viral proteome [68, 72, 74, 164], while others used computational predictions of MHC affinity to select small sets of peptides [68–71, 73]. Of these studies, only those which used multimeric MHC assays were able to unambiguously identify biological HLA restriction and the exact peptide determinants of a T cell response, whereas others used predicted or statistical assignments, sometimes within large peptide windows. To overcome the heterogeneity of this dataset, we binned the viral proteome into regions of 40 amino acids into which each study could contribute one or more identified epitope regions. A small number of recurrent epitope regions contained responses from three or more studies (Fig. 7B). Inspection of these recurrent regions broadly confirms the choice of S and N as particularly immunogenic proteins, likely due to their abundance, as well as one recurrent epitope region in the M protein. We also see strong recurrent responses to two regions of ORF3a, as well as three regions within non-structural proteins contained within ORF1ab (nsp3, nsp12, nsp13), which were not selected for consideration in our study. The identified recurrent epitope regions were strongly enriched for overlap with vaccine peptides selected in this study. In fact, 8/15 recurrent epitope regions in the S, M, and N proteins (and 8/20 total recurrent epitope regions) significantly overlapped at least one vaccine peptide. This degree of concordance gives us confidence that our computational selection process for T cell epitopes is at least to some degree predictive of biological SARS-CoV-2 T cell epitopes following infection.

### Murine validation of T and B cell epitope immunogenicity

**Fig. 8:**
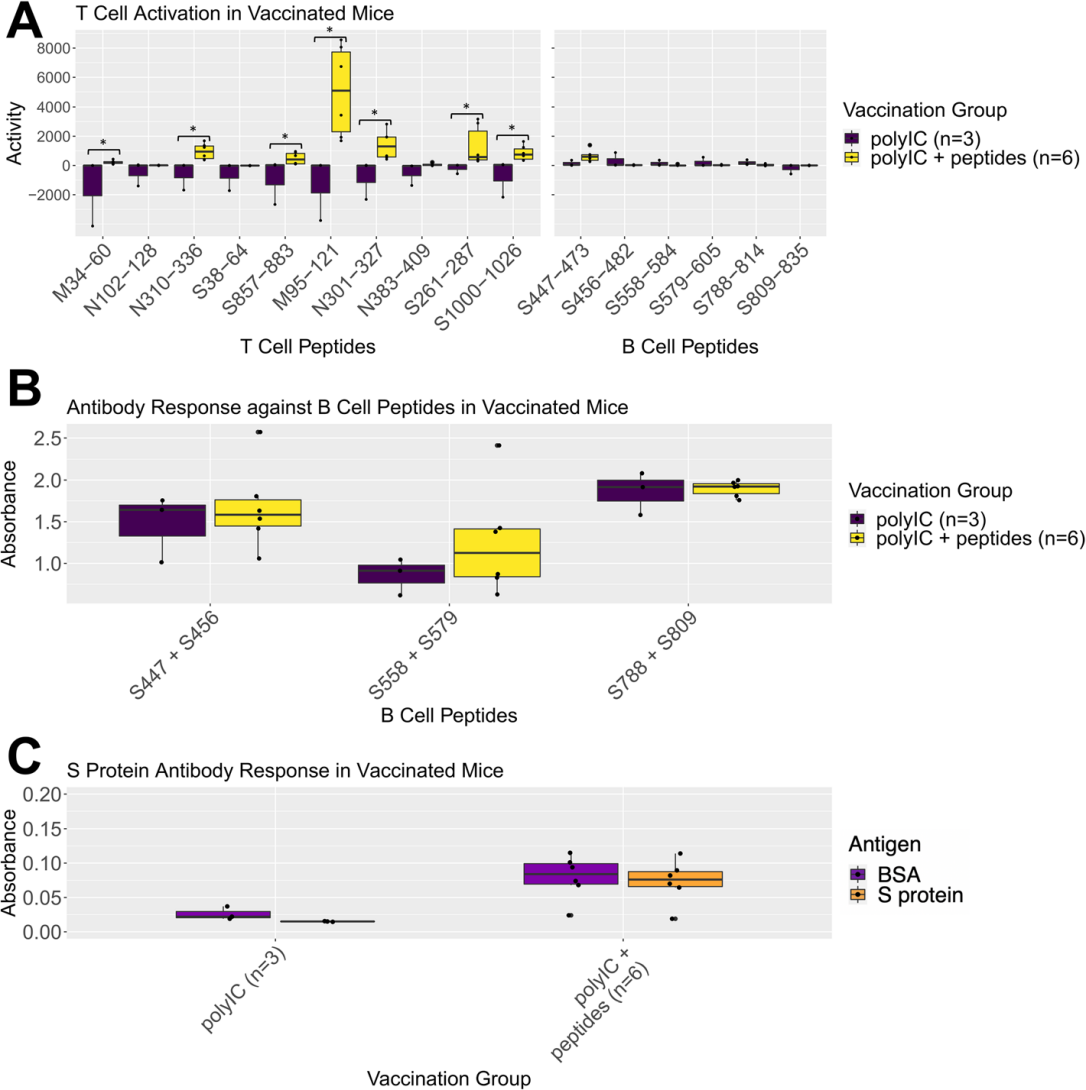
Experimental assessment of T and B cell epitope immunogenicity. **A** Mice were vaccinated with sixteen predicted T cell and B cell epitopes, designated as “peptides,” in combination with poly(I:C), or with poly(I:C) alone. T cell activity in response to vaccination was measured via IFN-y ELISpot with splenocytes isolated from mice at experimental day 21, plated with individual peptides. Activity was calculated by ELISpot plate reader. Peptide designations indicate protein, start, and end as shown in Fig. 6B. **B** Antibody response against predicted B cell peptide epitopes was measured via peptide ELISA. Wells were coated with pairs of predicted B cell peptides. **C** Antibody response against S protein was assessed via whole protein ELISA. Response to bovine serum albumin (BSA) was measured as negative control. For all subfigures, asterisks indicate statistically significant p value (< 0.05) from Mann-Whitney U tests of poly(I:C) + peptide groups compared to poly(I:C) alone

We sought to experimentally evaluate our minimum set of predicted T and B cell epitope candidates. We manually selected 16 of the 22 vaccine peptides for synthesis, keeping at most 2 peptides per overlapping region with a preference for those with predicted H2^d^ MHC ligands. We then vaccinated BALB/c mice with the 16 synthesized vaccine peptides and evaluated immune activation from humoral and T cell perspectives. Mice were vaccinated on experimental day 1, given booster vaccination on day 7, and sacrificed on day 21. We performed IFN-y ELISpot in order to assess T cell activation by culturing splenocytes from vaccinated animals alongside each of the peptides within the vaccine pool. We observed a statistically significant increase in IFN-y release in response to seven out of ten of our predicted T cell epitopes in mice vaccinated with peptides plus poly(I:C) versus poly(I:C) alone (Fig. 8A). We did not observe a statistically significant response against any of our six predicted B cell epitopes in our peptide vaccination group versus adjuvant alone. For evaluation of antibody responses, peptide (Fig. 8B) and S protein (Fig. 8C) ELISA from the day 21 sera of the above mice failed to show signal above adjuvant alone in all groups.

## Discussion

We report here a survey of the SARS-CoV-2 epitope landscape along with a strategy for prioritizing both T cell and B cell epitopes for vaccine development. Major vaccine efforts targeting coronaviruses have focused primarily on generation of neutralizing antibody responses [168–176]. CD4^+^ T cells provide help to B cells to support class switching, maturation, and antibody production. Additionally, they promote CD8^+^ T cell activation, maturation, and effector function. We therefore searched for vaccine peptide sequences which include both B cell epitopes and MHC ligands predicted to drive CD4^+^ and CD8^+^ T cell responses at high population frequencies within the U.S. based on data available in the first few months of the pandemic. Our current efforts are focused on testing the immunogenicity of these peptides in murine models, comparing those which contain overlapping and non-overlapping T and B cell epitopes. Results from such preclinical testing will inform an envisioned phase I clinical trial using a condensed peptide set targeting B cell epitopes with known viral neutralization plus optimal T cell epitopes.

Prior work has surveyed the epitope space of SARS-CoV-2 using analysis of sequence homology with SARS-CoV-1 epitopes, prediction of linear B cell epitopes, and prediction of T cell epitopes using IEDB tools. Grifoni et al. reported predicted T and B cell epitopes based on cross-referencing of known SARS epitopes with sequence homology to SARS-CoV-2 against SARS-CoV-2-specific parallel computational prediction [177]. This study did not consider epitope mapping of SARS-CoV-2 convalescent antibody repertoires, which may be important to achieve high specificity of B cell epitope predictions. Our prediction of T cell epitopes is conceptually similar to their computational process, but our study does not focus on conserved epitopes relative to SARS-CoV-1. Instead, we attempt to filter CD4^+^, CD8^+^, and B cell epitopes by additional considerations of vaccination suitability (e.g., polymorphism, accessibility) and go beyond epitope selection to vaccine peptides integrating different categories of epitopes. Ahmed et al. reported a set of predicted T and B cell SARS-CoV-2 epitopes with associated assay confirmation within the NIAID ViPR database. However, these predicted epitopes were largely limited to those with sequence homology between SARS-CoV-1 and SARS-CoV-2, given the paucity of available SARS-CoV-2 assay data in the spring of 2020. Several studies identified linear B cell epitopes on the SARS-CoV-2 surface glycoprotein from sera of viral exposed patients using peptide arrays [55, 56, 58] as well as phage immunoprecipitation sequencing (PhIP-Seq) [57]. These studies are an important source of information, but it has also been shown that antibodies which recognize peptides often cross-react primarily with proteins only in denatured conformations [178–180]. There is a risk that identified linear epitopes would not be able to promote viral neutralization in vivo due to a lack of surface exposure*.* Our work adds to this important emerging field by analyzing the SARS-CoV-2 HLA ligand landscape through binding affinity filters derived from validated IEDB HLA ligands, as well as deriving T and B cell vaccine candidates through rational filtering criteria grounded in SARS-CoV-2 biology, including predicted immunogenicity, epitope location, glycosylation sites, and polymorphic sites. Additionally, inclusion of corresponding murine epitopes allows for future studies to be performed in animal models of SARS-CoV-2. We expect the application of these filters will improve specificity of antiviral response.

Other computational methods for prediction of SARS-CoV-2 epitopes have been described [181–183] in a continuously growing body of literature. Many of these studies consider population-specific MHC allele frequencies and attempt to derive an optimal epitope set that allows for broad population coverage. Liu et al. [182] adds to this by further considering allelic linkage disequilibrium. Omnibus analysis of peptide-MHC binding from previously described tools was used to identify their peptide set comprising 19 each of MHC-I and MHC-II ligands. This method differs from our strategy in two ways: only considering peptide-MHC binding prediction rather than filtering for putative T cell epitopes, and deriving a set of 38 total minimal MHC-I and MHC-II ligands rather than identifying longer regions in the SARS-CoV-2 proteome that encompass regions with population-optimized T cell epitopes. While our capacity to predict peptide-MHC binding is reasonably accurate for MHC-I and variably accurate for MHC-II, our capacity to predict immunogenicity of any given minimal epitope remains limited. As such, we believe vaccinating with a longer (27mer) sequence containing multiple predicted minimal epitopes allows for a degree of purposeful imprecision, allowing for the optimal MHC-I and MHC-II sequences to be processed and presented in vivo. Compared to Poran et al. [181], which used a mass spectrometry-derived HLA presentation predictor, this peptide set is filtered through tetramer derived immunogenicity prediction—a more direct metric for epitope efficacy. Yarmarkovich et al. [183] addresses concerns for peptide immunogenicity versus autoimmunity by comparing predicted epitopes against a reference human peptidome.

While this study also filters for peptide overlap with self-epitopes, our immunogenicity prediction algorithm primarily considers peptide sequence features inspired by Calis et al. [146], predicted MHC scores, as well as the MHCflurry 2.0 [184] peptide processing score for CD8^+^ T cell epitopes, which are then used to fit a model against a validated viral tetramer dataset curated from IEDB [185]. Additionally, B cell epitopes were derived from in silico methods in Yarmarkovich et al., while this study used in vitro epitope mapping studies as the basis for our B cell epitope candidate set. Lastly, Gao et al. [186] approach the problem of SARS-CoV-2 epitope prediction by directly evaluating a candidate peptide’s sequence similarity to both the human proteome and the set of pathogenic epitopes in IEDB; based on the methodology, Luksza et al. [187] used for cancer neoantigen prediction. This approach is intrinsically limited by a hypothetical sequence homology between T cell epitopes in SARS-CoV-2 and previously identified pathogenic epitopes. On the other hand, we use a diverse set of peptide-MHC features and do not expect actual sequence homology with any existing known epitopes.

A key aspect of our epitope selection process is the prioritization of overlapping CD4^+^, CD8^+^, and B cell epitopes. As the role of T cell epitope vaccines in SARS-CoV-2 continues to be investigated in model systems, we furthermore cross-referenced human and murine T cell epitopes to allow for murine vaccine studies using human-relevant peptides in H2^b^ and H2^d^ haplotypes. We hypothesize that inclusion of CD8^+^ epitopes may allow for clearance of SARS-CoV-2 from infected cells, and the inclusion of CD4^+^ epitopes may allow for greater activation of both cytotoxic and humoral antiviral responses. Overlapping CD4+ and CD8+ epitopes allowed for selection of peptide candidates covering a large proportion of the population. We next attempted to identify candidates with overlapping CD4+/CD8+ epitopes with B cell epitopes. However, these candidate options were limited due to the paucity of predicted B cell candidates. Therefore, a more effective strategy would be to include overlapping CD4+/CD8+ optimized peptides together with separate B cell optimized peptides. We expect this to provide the most robust and broad antiviral adaptive immune coverage by activating CD4+ T cells, CD8+ T cells, and B cells.

To this end, we predicted and tested the immunogenicity of peptides optimized for both overlapping CD4^+^/CD8^+^ T cell epitopes as well as peptides optimized for B cell epitopes. We observed statistically significant T cell activation measured by IFN-y release in response to seven of our 10 predicted T cell stimulatory epitopes when administered with poly(I:C) adjuvant as compared to vaccination with poly(I:C) alone. None of our six predicted B cell epitopes generated significant T cell activation, indicating that our method for predicting T cell immunogenicity is appropriately specific. A 70% success rate for prediction of T cell epitopes that would activate T cells to generate significantly enhanced IFN-y release demonstrates that our computational prediction of peptide vaccines was successful from a T cell standpoint. Further studies to assess (1) the CD4+ versus CD8+ responses against each peptide, (2) immunogenicity of individual epitopes within each peptide, and (3) the protective capacities of these epitopes are required to validate their therapeutic potential.

Contrasting these T cell findings, we did not observe increased antibody response against any of our predicted B cell epitopes in peptide-vaccinated mice compared to those vaccinated with adjuvant alone. We also did not observe any significant antibody response against S protein above negative control in vaccinated mice. This indicates that while our strategy was successful in predicting immunogenic T cell epitopes, our predicted B cell epitopes did not provide robust B cell activation by day 21. Options to further investigate these results include titrating dosage of the administered B cell peptides to evaluate whether concentrations used were sufficient to generate robust antibody responses, or further refinement of our criteria for B cell epitope prediction in order to predict epitopes more likely to generate an antibody response. Whether T cell responses in absence of antibody responses are sufficient for antiviral protection remains unclear and can be addressed in future viral challenge studies.

In addition to epitope selection, optimal adjuvant choice for a SARS-CoV-2 vaccine is currently unclear. Prior evidence from SARS-CoV-1 suggested a Th2 dominant response to be associated with worse outcomes [13]—thus, adjuvant selection may also play an important role in SARS-CoV-2 in skewing the helper arm toward a Th1 phenotype. Patients with severe COVID-19 demonstrate elevated levels of CCR6+ Th17 cells [188]. Additionally, many COVID-19 patients with acute respiratory distress syndrome (ARDS) demonstrated cytokine storm manifested by elevation of a variety of cytokines, of which several are involved in Th17 responses [189]. In MERS patients, increased IL-17 to type I IFN is associated with worse outcome [190]. Altogether, the Th17 response may contribute to increased risk of severe pulmonary injury and worse outcomes in COVID-19 patients [191]. As the Th1 and Th17 cellular response pathways are closely linked, co-therapies that inhibit Th17 activation (e.g., secukinumab, tocilizumab) have been proposed for use in COVID-19; however, the efficacy of these therapies remains to be seen. The role of other helper subsets (Th9, Th18) remains even more poorly understood. Relevant for the vaccine studies presented here, poly(I:C) appears to primarily activate Th1 cells, skewing the immune response toward a phenotype that may be most beneficial [192]. Further studies would be needed to assess which subtypes of T cells were activated by our vaccine formulations.

One limitation of our study is that, while we use epitope mapping data with direct biological evidence for B cell epitopes in SARS-CoV-2, the T cell epitopes we report were all derived from computational prediction. In an effort to partially overcome this weakness, we applied binding affinity and immunogenicity prediction filters grounded in validated IEDB binding and tetramer studies. Other filtering criteria for T cell epitopes have been evaluated, including allergenicity, antigenicity, stability, and inflammatory/cytotoxic response [193–195]; it remains to be seen if these or other filtering criteria improve T cell epitope selection in SARS-CoV-2. Reassuringly, our selection of T cell-directed vaccine peptides demonstrates significant overlap with the recurrent epitopes identified in eight different studies examining T cell responses in COVID-19 patients (Fig. 7). Le Bert et al. looked for T cell epitopes within the nucleocapsid (N), nsp7 and nsp13 proteins in PBMCs of recovered COVID-19 patients using an IFN-γ ELISpot assay [196]. They identified two recurrent epitope regions (N101-120, N321-340) which overlap with multiple 27mer vaccine peptides in this paper (Fig. 6B, peptides 4–8). Shomuradova et al. also identified COVID-19 patient T cell epitopes, but using A*02:01 tetramers loaded with 13 distinct peptides from the surface glycoprotein (S) [71]. Two of these 13 peptides showed recurrent reactivity across 14 A*02:01-positive patients (S269-277 and S1000-1008). Both of these epitopes are also included in multiple 27mer vaccine peptides (Fig. 6B, peptides 11 and 15). Across all eight studies considered, the most recurrently identified epitopes fall within two regions, both in the nucleocapsid protein (N) around positions N100 and N300 (Fig. 7B), overlapping with multiple vaccine peptides selected by our algorithm. It is worth noting that our heuristic for selecting abundant proteins (only considering epitopes and vaccine peptides from the M, N, and S proteins) was moderately successful in that 15/20 recurrent epitope regions occurred in these proteins. While we missed recurrent epitope regions in ORF3a, nsp3, nsp12, and nsp13, filtering our predictions to the most abundant proteins allowed us to avoid many false positive predictions from ORF1ab and perform much better in predicting true T cell epitopes.

It is worth noting that the dataset of biologically measured T cell responses to SARS-CoV-2 infection which we curated to evaluate our vaccine peptide selection overlaps significantly with another study by Quadeer et al. [197]. The biggest difference between their approach and ours is that we do not require HLA restriction of identified epitope regions and can thus use a larger number of epitopes from assays such as unbiased ELISPOT screening. Since our evaluation seeks primarily to ascertain whether our vaccine peptides are highly enriched for immunogenic epitopes, we are less stringent in knowing exactly which epitopes are present and to which HLA alleles they bind.

A different potential limitation of this study is the insensitivity of our experiments to the total potential space of SARS-CoV-2 antibody epitopes. Our B cell epitope analyses start with only 58 identified linear antibody epitopes on the surface glycoprotein of SARS-CoV-2, while it is likely that many other epitopes are possible. Second, these linear epitope mappings do not allow for identification of antibodies which bind tertiary/quaternary protein structures. Lastly, identification of epitopes via array studies depended on differences in antibody binding to potential linear epitopes between uninfected and infected persons. There may be some cross-reactivity between antibodies generated against other coronaviruses and SARS-CoV-2, which if present might show reactivity in our screening assay. If true, our strategy would not identify these epitopes as specific for SARS-CoV-2. Similarly, we excluded viral regions with significant polymorphism across the viral population. We instead focused on conserved regions of SARS-CoV-2 to identify epitopes that would be most broadly targetable in the human population. For these reasons, we do not present our antibody data as describing the complete set of SARS-CoV-2 epitopes.

## Conclusions

Our study sought to design a peptide vaccine for SARS-CoV-2 targeting immune responses from B cells, CD4^+^ T cells, and CD8^+^ T cells. This kind of vaccine may be a useful addition to the evolving landscape of SARS-CoV-2 vaccines since its rapid manufacturing and precise design may help fill gaps in immunity that arise due to antigenic drift of new viral variants. However, we emphasize that epitope selection is only one aspect of the problem, and a key question is whether a peptide vaccine can be sufficiently immunogenic. Adjuvant selection, conjugation to carriers such as KLH [43] or rTTHC [198], and prime/boost approaches using orthogonal platforms are all potential avenues to explore. Thus far, we have demonstrated the immunogenic capacity of our T cell epitope selection process coupled with linear peptide vaccination using poly(I:C) as an adjuvant. It is possible that the selected B cell epitopes in this work may still be useful for eliciting neutralizing responses when encoded using a more conformationally stable immunogen. We anticipate that the sets of vaccine peptides reported here may be valuable in the preclinical development of these approaches.

## Supplementary Information

**Additional file 1.** Contains all supplemental figures (Fig. S1 - S10).

**Additional file 2: Table S1.** All SARS-CoV-2 MHC-I ligands contained in the top 5% of U.S. HLA alleles. **Table S2.** All SARS-CoV-2 MHC-II ligands contained in the top 5% of U.S. HLA alleles. **Table S3.** All SARS-CoV-2 MHC-I ligands contained in the top 5% of worldwide HLA alleles. **Table S4.** All SARS-CoV-2 MHC-II ligands contained in the top 5% of worldwide HLA alleles. **Table S5.** Summary of SARS-CoV-1 MHC ligands previously described in the literature. **Table S6.** SARS-CoV-2 B cell linear epitopes from array/mapping studies. **Table S7.** SARS-CoV-2 T cell epitopes within S, M, and N proteins. **Table S8.** SARS-CoV-2 T cell epitopes within all proteins. **Table S9.** Curated a dataset of published T cell epitope mapping studies.

## Data Availability

The datasets generated and/or analysed during the current study are available in the Vincent lab github repository, https://github.com/Benjamin-Vincent-Lab/Landscape-and-Selection-of-Vaccine-Epitopes-in-SARS-CoV-2 [199]. Several data files larger than 100 Mb and supplemental tables are available at https://data.mendeley.com/datasets/c6pdfrwxgj/6 [200].

## Abbreviations

RBD: Receptor binding domain
ACE2: Angiotensin-converting enzyme 2
IEDB: Immune Epitope Database
PhIP-Seq: Phage immunoprecipitation sequencing
EL: Elution ligand
BA: Binding affinity
S: Surface glycoprotein
N: Nucleocapsid protein
M: Membrane
FP: Fusion peptide
HR: Heptad repeat
RBM: Receptor binding motif
