# Supplementary material for: Landscape and selection of vaccine epitopes in SARS-CoV-2
Source: Genome Med. 2021 Jun 14;13:101. doi: 10.1186/s13073-021-00910-1 (PMC8201469; doi:10.1186/s13073-021-00910-1)
Supplement: Supplementary file 1 — Additional file 1. Contains all supplemental figures (Fig. S1 - S10). [file 13073_2021_910_MOESM1_ESM.pdf]

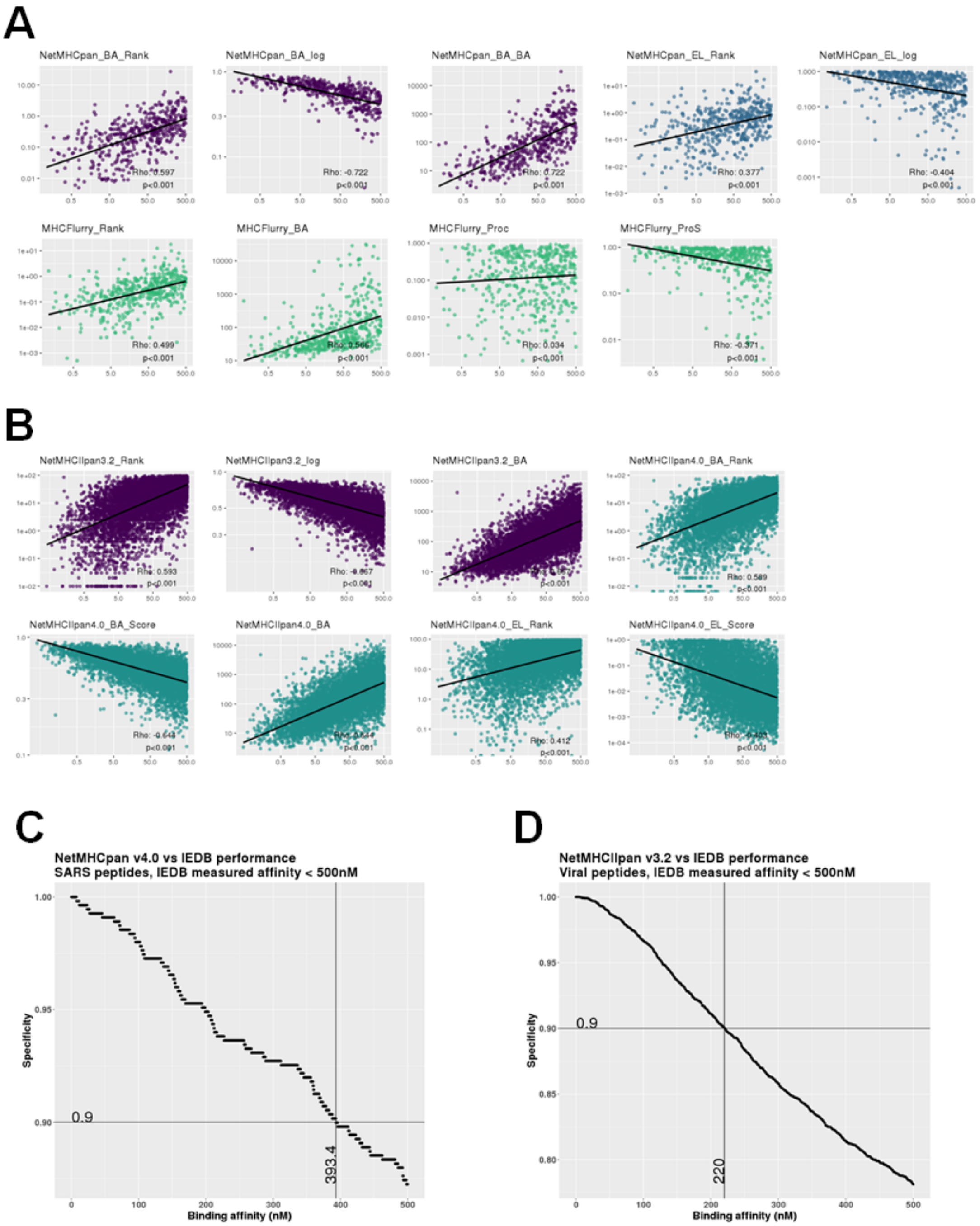

Fig. S1: Selection criteria for predicted HLA ligands. ***(A&B)*** *Scatterplot of IEDB binding affinity (x-axis) versus predicted features (y-axis) for HLA-I SARS ligands* ***(A)*** *and HLA-II viral ligands* ***(B)****, with linear fit and Spearman correlation represented. Color represents the prediction tool used for each feature.* ***(C&D)*** *Plot of NetMHCpan 4.0* ***(C)*** *and NetMHCIIpan 3.2* ***(D)*** *binding affinity (x-axis) versus specificity (y-axis) for predicting binding ligand, as defined by IEDB binding affinity < 500 nM.*

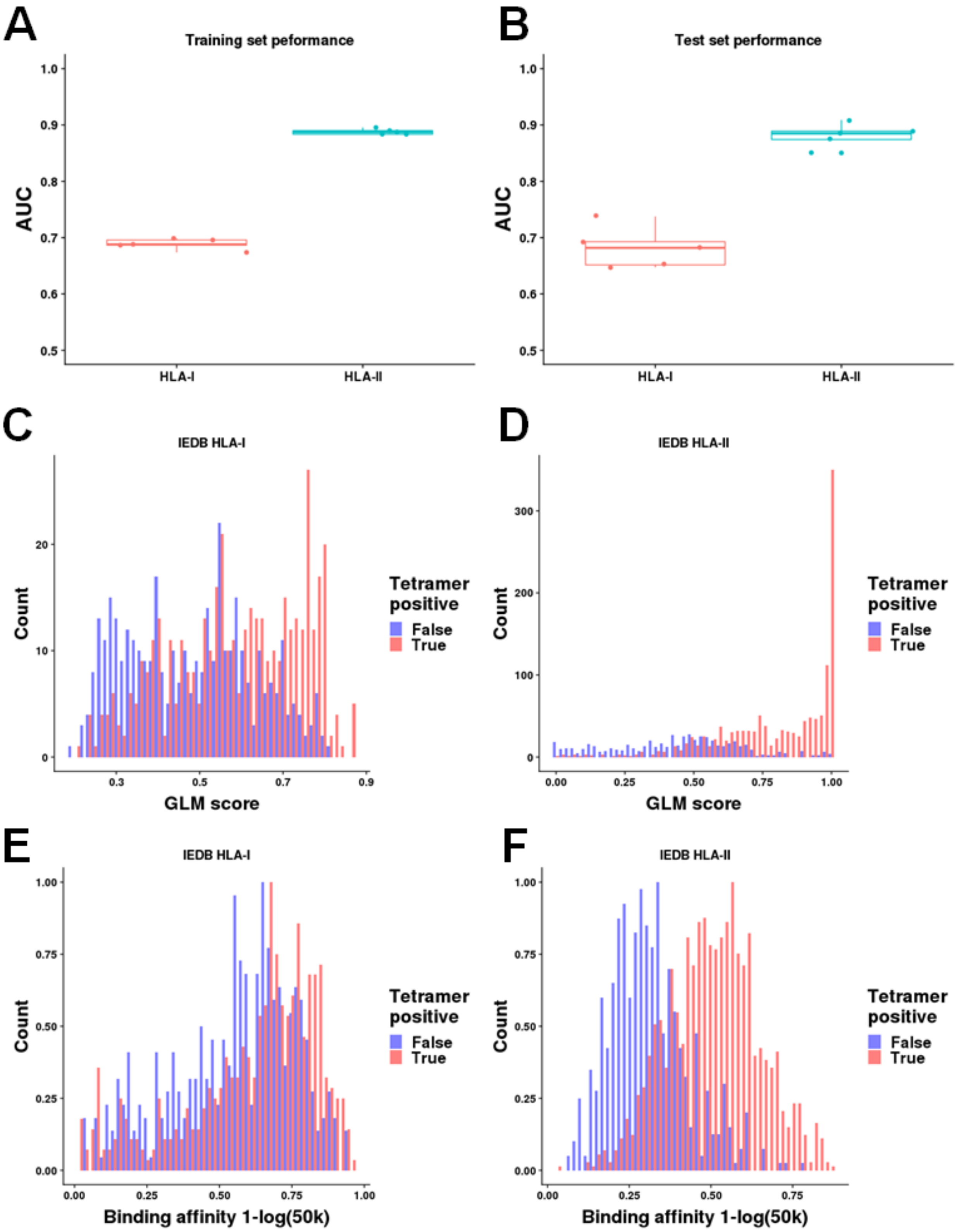

Fig. S2: Summary of multivariable GLM model for prediction of epitope immunogenicity, trained on IEDB tetramer data. ***(A&B)*** *Area under the curve of HLA-I (red) and HLA-II (blue) GLM models for 5-fold cross validation training* ***(A)*** *and test* ***(B)*** *sets.* ***(C&D)*** *Histograms of GLM scores for tetramer positive (red) and negative (blue) $CD8^+$* ***(C)*** *and $CD4^+$* ***(D)*** *epitopes in IEDB tetramer dataset.* ***(E&F)*** *Histograms of binding affinity scores for tetramer positive (red) and negative (blue) $CD8^+$* ***(E)*** *and $CD4^+$* ***(F)*** *epitopes in IEDB tetramer dataset.*

A

```
Deviance Residuals:
    Min       1Q   Median       3Q      Max
-1.8262  -1.0980   0.6825   1.0449   1.7833

Coefficients:
                  Estimate Std. Error z value Pr(>|z|)
(Intercept)        -0.7444     0.2108  -3.531 0.000413 ***
Flurry_proc_score   1.0901     0.3146   3.465 0.000530 ***
EL_Score            1.4007     0.2876   4.870 1.11e-06 ***
Binding_affinity    3.1258     0.8026   3.894 9.84e-05 ***
Small              -1.1527     0.4761  -2.421 0.015457 *
---
Signif. codes:  0 '***' 0.001 '**' 0.01 '*' 0.05 '.' 0.1 ' ' 1

(Dispersion parameter for binomial family taken to be 1)

    Null deviance: 1117.2  on 808  degrees of freedom
Residual deviance: 1025.9  on 804  degrees of freedom
AIC: 1035.9

Number of Fisher Scoring iterations: 4
```

B

```
Deviance Residuals:
    Min       1Q   Median       3Q      Max
-4.1923  -0.6214   0.1554   0.7503   5.0196

Coefficients:
                 Estimate Std. Error z value Pr(>|z|)
(Intercept)        1.4417     0.2348   6.139 8.30e-10 ***
EL_Score           9.5627     0.9015  10.608  < 2e-16 ***
Binding_affinity -17.5174     2.0503  -8.544  < 2e-16 ***
Cyclic            -5.2677     1.2746  -4.133 3.59e-05 ***
Aromatic          -2.7801     0.9348  -2.974  0.00294 **
Acidic            -2.2112     0.8657  -2.554  0.01065 *
Basic             -1.4255     0.7661  -1.861  0.06278 .
---
Signif. codes:  0 '***' 0.001 '**' 0.01 '*' 0.05 '.' 0.1 ' ' 1

(Dispersion parameter for binomial family taken to be 1)

    Null deviance: 2285.8  on 1859  degrees of freedom
Residual deviance: 1504.4  on 1853  degrees of freedom
AIC: 1518.4
```

Fig. S3: (A&B) *HLA-I* ***(A)*** *and HLA-II* ***(B)*** *GLM predicting for tetramer positivity as a function of binding and amino acid features.*

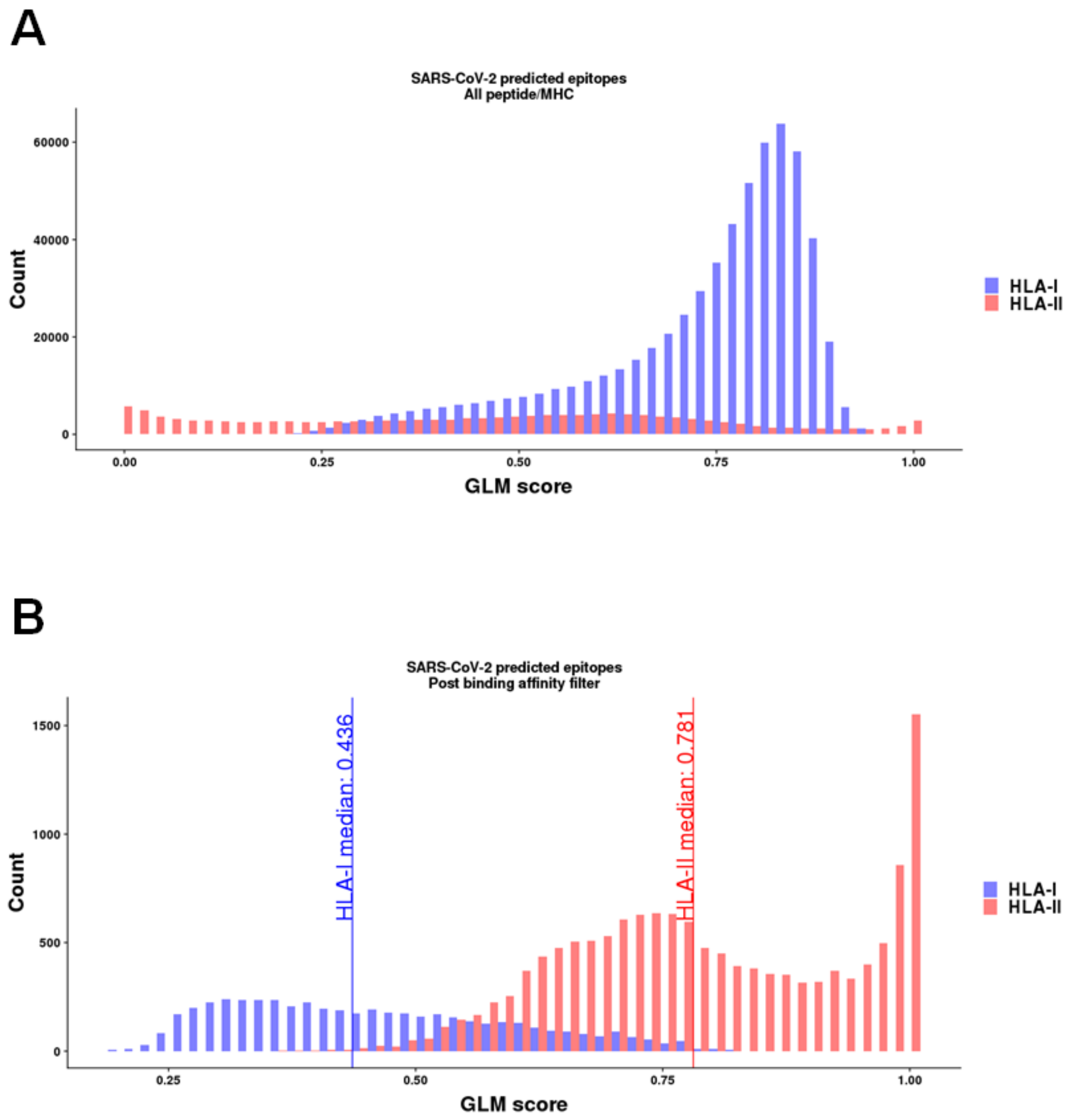

Fig. S4: (A&B) *Distribution of GLM scores among predicted SARS-CoV-2 T cell epitopes prior to binding affinity filter* ***(A)*** *and after binding affinity filter* ***(B)****. Vertical lines in* ***(B)*** *represent median GLM score for predicted CD4*$^{+}$ *and CD8*$^{+}$ *epitopes.*

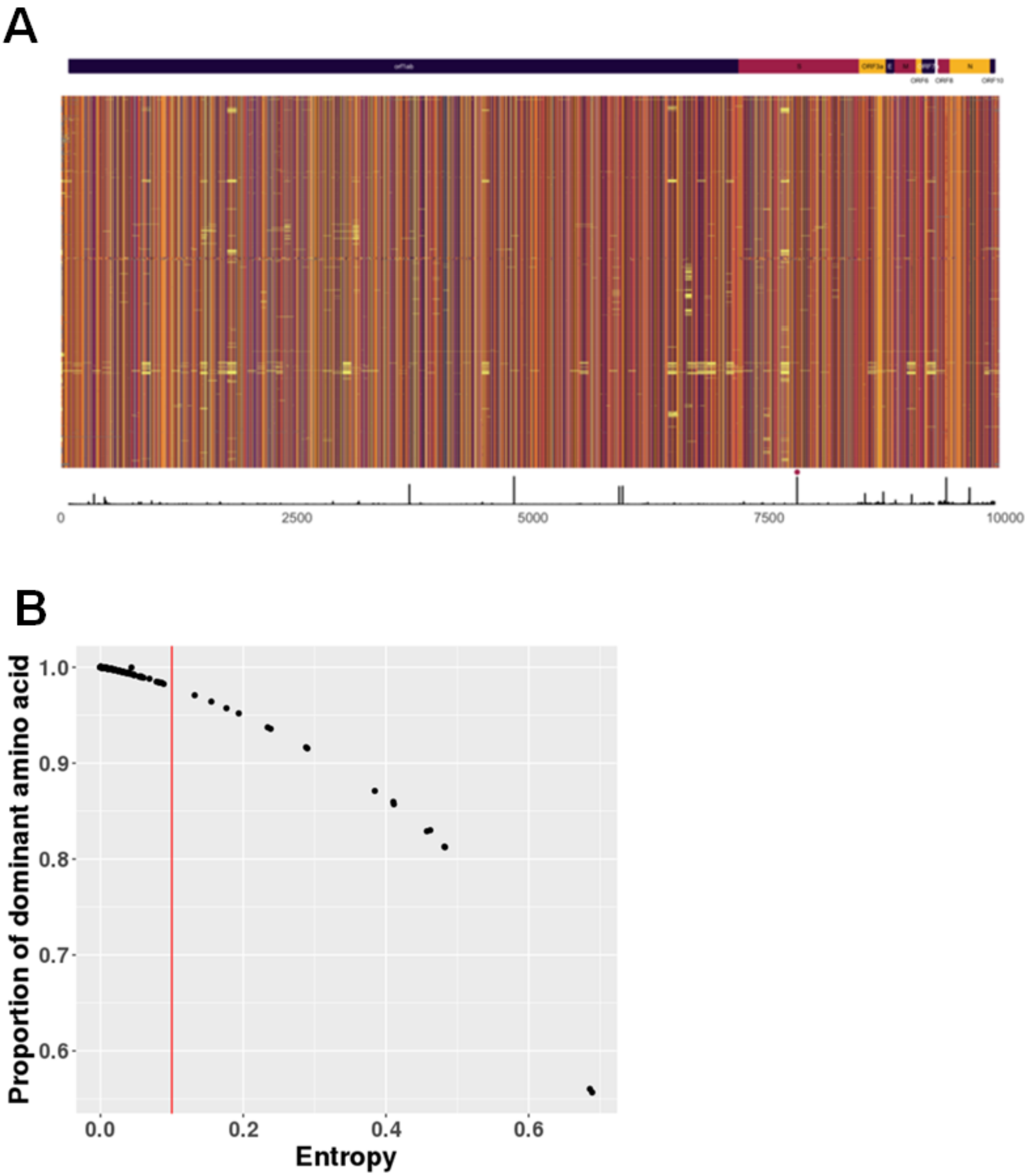

Fig. S5: Sequence level variation across SARS-CoV-2 viral proteomes in the GISAID database. ***(A)*** *Locations along the viral genome represented by x-axis, with individual genomes (n=7881) along the y-axis. Colors represent amino acid residues (plotted on viridis "inferno" color scheme, dark (A) to light (Y) in alphabetical order of amino acid letter abbreviations; gap/unknown = grey), aligned using Augur/MAFFT derived multiple sequence alignment (MSA; see Methods: SARS-CoV-2 entropy calculations). The histogram along the y-axis represents entropy at each location, with position 614 of the S protein marked with a red dot. Proteins by locations are shown by the column-side colorbar.* ***(B)*** *Entropy versus proportion of the dominant amino acid residue by position along MSA-aligned genomes, with the red line representing an entropy cutoff of 0.1 used for this study.*

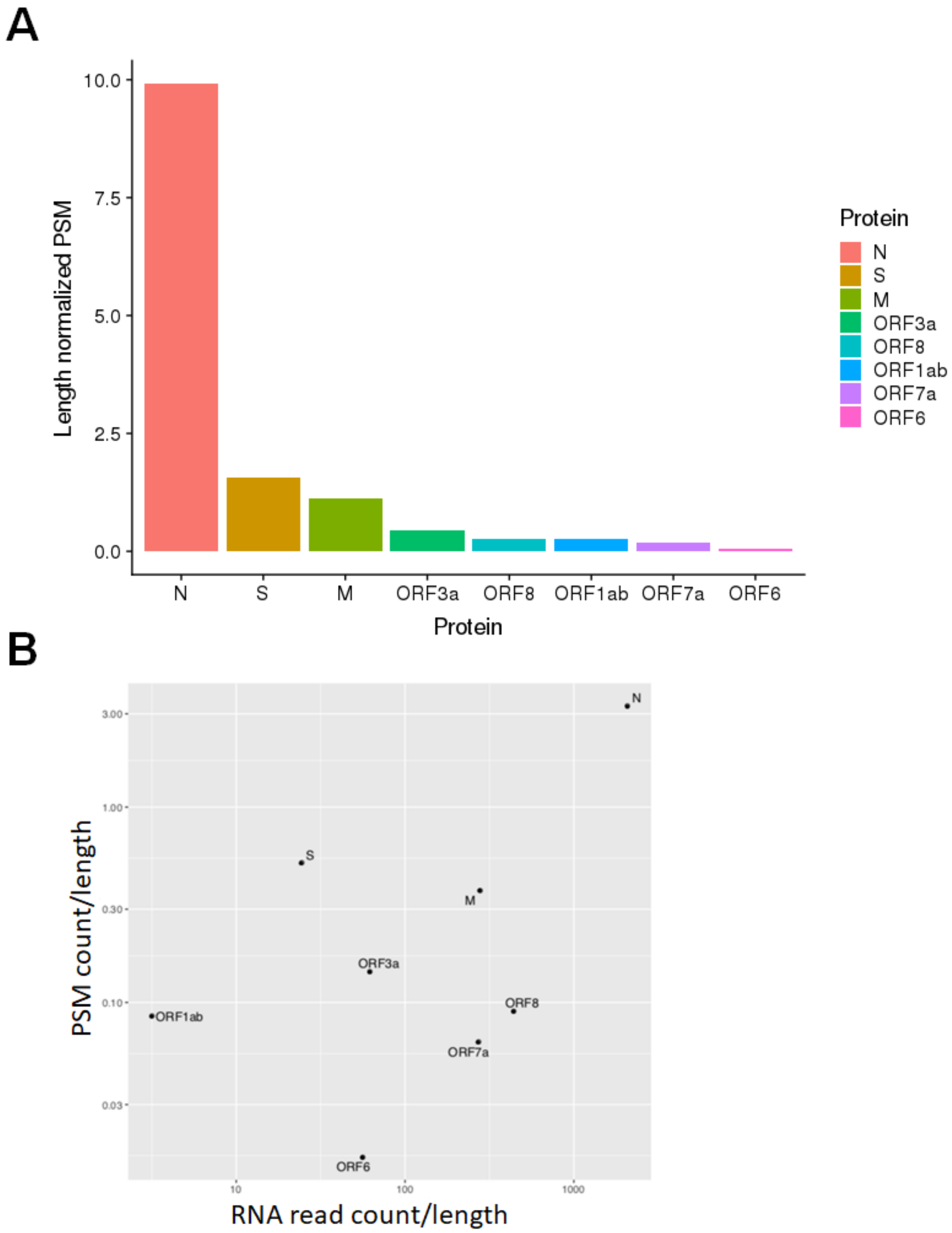

***Fig. S6:* (A)** *Length normalized peptide spectrum match (PSM) counts for SARS-CoV-2 proteins.* **(B)** *Length normalized PSM versus length normalized RNA-seq read counts for SARS-CoV-2 proteins.*

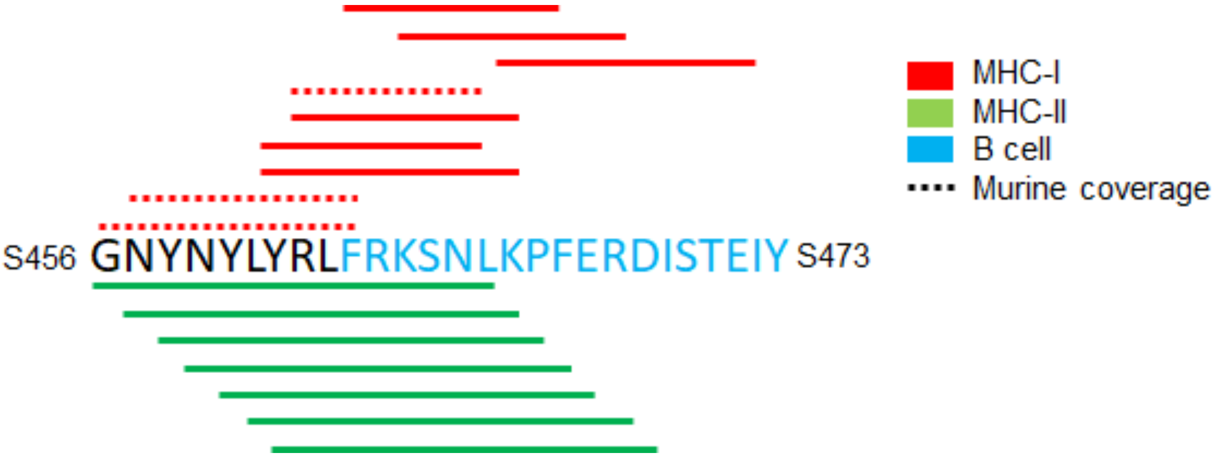

Fig. S7: *Representation of 27mer peptides, containing predicted SARS-CoV-2 CD8⁺ T cell (red), CD4⁺ T cell (green), and B cell (blue) epitopes. Overlapping murine MHC ligands are represented by dashed lines.*

A

Peptide Feature	Difficulty Score
Entire peptide hydrophobic (GRAVY score > 2.0)	1
Difficult N-terminal residue	1
Difficult C-terminal residue	2
Number of cysteine or methionine residues	2
Difficult local hydrophobicity (local GRAVY score > 1.5)	2
Moderately unstable di-peptides	3
Disulfide bonds (more than one cysteine)	5
Extreme local hydrophobicity	10
Extremely unstable di-peptides	10

B

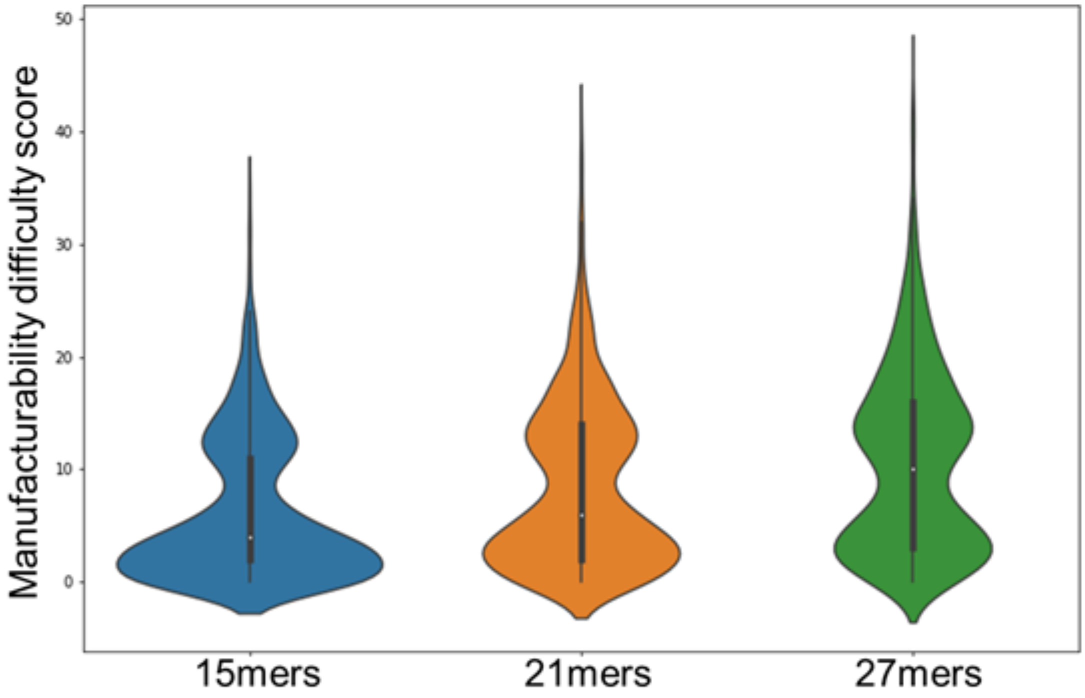

Fig. S8:* (A)** *Manufacturability difficulty scoring criteria for vaccine peptide candidates.* ***(B) *Distribution of manufacturability difficulty scores for 15mer, 21mer, and 27mer peptide sets.*

A

Symbol	Set	# Peptides	HLA-I Coverage	HLA-II Coverage	Total Coverage	# B-cell Epitope Regions
⊛	CD4+/CD8+	5	90.6%	88.5%	80.2%	0
⊛^{d}	CD4+/CD8+ (H2^{d} ligands)	3	81.1%	76.2%	61.8%	0
⊛^{b}	CD4+/CD8+ (H2^{b} ligands)	3	81.8%	62.5%	51.1%	0
⊛^{bd}	CD4+/CD8+ (H2^{b} and H2^{d} ligands)	2	77.2%	65.8%	50.8%	0
○	CD4+	3	83.9%	88.5%	74.3%	0
○^{d}	CD4+ (H2^{d} ligands)	3	86.7%	84.7%	73.4%	0
○^{b}	CD4+ (H2^{b} ligands)	3	83.9%	84.7%	71.1%	0
○^{bd}	CD4+ (H2^{b} and H2^{d} ligands)	3	86.7%	84.7%	73.4%	0
*	CD8+	3	95.8%	38.4%	36.8%	0
*^{d}	CD8+ (H2^{d} ligands)	3	94.6%	22.6%	21.4%	0
*^{b}	CD8+ (H2^{b} ligands)	3	91.2%	46.5%	42.4%	0
*^{bd}	CD8+ (H2^{b} and H2^{d} ligands)	3	91.2%	46.5%	42.4%	0
⊛⃞	B-Cell/CD4+/CD8+	4	77.2%	45.8%	35.3%	3
⧇	B-Cell/CD4+	5	77.2%	62.7%	48.4%	3
⧇^{b}	B-Cell/CD4+ (H2^{b} ligands)	2	0.0%	39.4%	0.0%	2
⧆	B-Cell/CD8+	6	84.2%	29.9%	25.2%	3
⧆^{d}	B-Cell/CD8+ (H2^{d} ligands)	1	77.2%	20.4%	15.8%	1
⧆^{b}	B-Cell/CD8+ (H2^{b} ligands)	2	72.5%	20.4%	14.8%	1
⧆^{bd}	B-Cell/CD8+ (H2^{b} and H2^{d} ligands)	1	77.2%	20.4%	15.8%	1
□	B-Cell	3	44.0%	11.9%	5.2%	3

B

	Sequence	Protein	Start	End	B-cell Epitope Region	HLA-I Coverage	HLA-II Coverage	H2^{b} I	H2^{b} II	H2^{d} I	H2^{d} II	Selection Sets
1	LLQFAYANRNRFLYI	M	34	48		77.0%	36.0%	+	+	+	+	○ ○^{b} ○^{d}○^{bd} ⊛ ⊛^{d} ⊛^{bd}
2	YANRNRFLYIIKLIF	M	39	53		78.0%	0.0%	+	-	+	-	*^{d}
3	ANRNRFLYIIKLIFL	M	40	54		81.0%	0.0%	+	-	+	-	*^{b}*^{bd}
4	YFIASFRLFARTRSM	M	95	109		78.0%	20.0%	+	-	+	+	*
5	SFRLFARTRSMWSFN	M	99	113		73.0%	46.0%	+	+	-	+	⊛^{b}
6	LSPRWYFYYLGTGPE	N	104	118		49.0%	0.0%	+	-	+	-	*^{d} *^{b} *^{bd}
7	ATKAYNVTQAFGRRG	N	264	278		24.0%	46.0%	+	+	+	-	⊛^{b}
8	PQIAQFAPSASAFFG	N	302	316		17.0%	39.0%	-	+	+	+	○^{d} ○^{bd} ⊛^{d}
9	SASAFFGMSRIGMEV	N	310	324		56.0%	37.0%	+	-	+	-	⊛
10	MEVTPSGTWLTYTGA	N	322	336		46.0%	0.0%	-	-	-	-	*
11	PSGTWLTYTGAIKLD	N	326	340		14.0%	52.0%	+	+	-	-	○^{b}
12	QQTVTLLPAADLDDF	N	389	403		11.0%	34.0%	-	-	-	-	○⊛
13	IGINITRFQTLLALH	S	231	245		61.0%	62.0%	+	-	+	+	⊛⊛^{d}
14	YYVGYLQPRTFLLKY	S	265	279		88.0%	23.0%	-	+	+	-	**^{d}
15	LTDEMIAQYTSALLA	S	865	879		42.0%	46.0%	+	+	+	+	*^{b} *^{bd} ⊛^{b}⊛^{bd}
16	RAAEIRASANLAATK	S	1014	1028		30.0%	79.0%	-	+	-	+	○ ○^{b} ○^{d}○^{bd} ⊛
17	GGNYNYLYRLFRKSN	S	446	460	456-FRKSNLKPFERDISTEIY-473	37.0%	20.0%	+	-	+	-	⧆
18	NYNYLYRLFRKSNLK	S	448	462	456-FRKSNLKPFERDISTEIY-473	77.0%	20.0%	+	-	+	-	⧆^{d} ⧆^{bd} ⧇⊛⃞
19	YNYLYRLFRKSNLKP	S	449	463	456-FRKSNLKPFERDISTEIY-473	73.0%	20.0%	+	-	-	-	⧆^{b}
20	YLYRLFRKSNLKPFE	S	451	465	456-FRKSNLKPFERDISTEIY-473	73.0%	20.0%	+	-	-	-	⧆
21	YRLFRKSNLKPFERD	S	453	467	456-FRKSNLKPFERDISTEIY-473	73.0%	23.0%	+	-	-	-	⧇⊛⃞
22	RLFRKSNLKPFERDI	S	454	468	456-FRKSNLKPFERDISTEIY-473	56.0%	0.0%	+	-	-	-	⧆^{b}
23	FRKSNLKPFERDIST	S	456	470	456-FRKSNLKPFERDISTEIY-473	32.0%	0.0%	-	-	-	-	⧆
24	KSNLKPFERDISTEI	S	458	472	456-FRKSNLKPFERDISTEIY-473	29.0%	0.0%	-	-	-	-	□
25	LKPFERDISTEIYQA	S	461	475	456-FRKSNLKPFERDISTEIY-473	20.0%	12.0%	-	-	-	-	⧆
26	ISTEIYQAGSTPCNG	S	468	482	456-FRKSNLKPFERDISTEIY-473	0.0%	21.0%	-	+	-	-	⧇⧇^{b}
27	ADTTDAVRDPQTLEI	S	570	584	580-QTLE-583	0.0%	0.0%	-	-	-	-	□ ⧇ ⊛⃞
28	PQTLEILDITPCSFG	S	579	593	580-QTLE-583	13.0%	0.0%	-	-	-	-	⧆
29	GFNFSQILPDPSKPS	S	799	813	809-PSKP-812	0.0%	23.0%	-	+	-	-	⧇⧇^{b}
30	FNFSQILPDPSKPSK	S	800	814	809-PSKP-812	21.0%	12.0%	-	-	-	-	□ ⧆ ⊛⃞

Fig. S9: T cell and B cell vaccine candidates. (A) *15mer vaccine peptide sets selecting for best CD4^{+}, CD8^{+}, CD4^{+}/CD8^{+}, and B cell epitopes with HLA-I, HLA-II, and total population coverage.* ***(B)*** *Unified list of all selected 15mer vaccine peptides. Vaccine peptides containing predicted ligands for murine MHC alleles (H2-b and H2-d haplotypes) are indicated in their respective columns.*

A

Symbol	Set	# Peptides	HLA-I Coverage	HLA-II Coverage	Total Coverage	# B-cell Epitope Regions
$\circledast$	CD4+/CD8+	3	84.9%	84.7%	71.9%	0
$\circledast^{d}$	CD4+/CD8+ (H2^{d} ligands)	4	90.2%	84.7%	76.4%	0
$\circledast^{b}$	CD4+/CD8+ (H2^{b} ligands)	4	93.9%	84.7%	79.5%	0
$\circledast^{bd}$	CD4+/CD8+ (H2^{b} and H2^{d} ligands)	4	92.1%	84.7%	78.0%	0
$\circ$	CD4+	3	92.2%	88.5%	81.6%	0
$\circ^{d}$	CD4+ (H2^{d} ligands)	3	92.2%	88.5%	81.6%	0
$\circ^{b}$	CD4+ (H2^{b} ligands)	3	69.5%	84.7%	58.9%	0
$\circ^{bd}$	CD4+ (H2^{b} and H2^{d} ligands)	3	93.8%	84.7%	79.4%	0
$\ast$	CD8+	3	95.1%	62.2%	59.1%	0
$\ast^{d}$	CD8+ (H2^{d} ligands)	3	94.7%	68.9%	65.3%	0
$\ast^{b}$	CD8+ (H2^{b} ligands)	3	94.7%	68.9%	65.3%	0
$\ast^{bd}$	CD8+ (H2^{b} and H2^{d} ligands)	3	94.7%	68.9%	65.3%	0
$\boxed{\circledast}$	B-Cell/CD4+/CD8+	4	84.2%	62.7%	52.8%	3
$\boxed{\circ}$	B-Cell/CD4+	4	84.2%	62.7%	52.8%	3
$\boxed{\circ}^{b}$	B-Cell/CD4+ (H2^{b} ligands)	2	37.2%	39.4%	14.6%	2
$\boxed{\ast}$	B-Cell/CD8+	3	88.9%	22.6%	20.1%	3
$\boxed{\ast}^{d}$	B-Cell/CD8+ (H2^{d} ligands)	1	77.2%	38.4%	29.7%	1
$\boxed{\ast}^{b}$	B-Cell/CD8+ (H2^{b} ligands)	2	87.2%	22.6%	19.7%	2
$\boxed{\ast}^{bd}$	B-Cell/CD8+ (H2^{b} and H2^{d} ligands)	1	77.2%	38.4%	29.7%	1
$\square$	B-Cell	3	78.0%	40.7%	31.8%	3

B

	Sequence	Protein	Start	End	B-cell Epitope Region	HLA-I Coverage	HLA-II Coverage	H2^{b} I	H2^{b} II	H2^{d} I	H2^{d} II	Selection Sets
1	LLQFAYANRNRFLYIIKLIFL	M	34	54		89.0%	36.0%	+	+	+	+	$\ast\ \ast^{b}\ \ast^{d}\ \ast^{bd}$ $\circ\ \circ^{d}\ \circ^{bd}\ \circledast^{d}$ $\circledast^{b}\ \circledast^{bd}$
2	FVLAAVYRINWITGGIAIAMA	M	65	85		42.0%	76.0%	+	+	-	+	$\circ^{b}\ \circledast\ \circledast^{b}$
3	LSYFIASFRLFARTRSMWSFN	M	93	113		78.0%	46.0%	+	+	+	+	$\circledast^{bd}$
4	LSPRWYFYYLGTGPEAGLPYG	N	104	124		49.0%	23.0%	+	+	+	-	$\ast$
5	GTRNPANNAAIVLQLPQGTTL	N	147	167		20.0%	55.0%	-	+	-	+	$\circ^{bd}$
6	IAQFAPSASAFFGMSRIGMEV	N	304	324		63.0%	51.0%	+	+	+	+	$\circledast^{d}\ \circledast^{bd}$
7	SASAFFGMSRIGMEVTPSGTW	N	310	330		65.0%	37.0%	+	-	+	-	$\ast^{b}\ \ast^{d}\ \ast^{bd}$
8	IGMEVTPSGTWLTYTGAIKLD	N	320	340		54.0%	52.0%	+	+	-	-	$\circledast^{b}$
9	GTWLTYTGAIKLDDKDPNFKD	N	328	348		26.0%	62.0%	+	+	-	-	$\circ^{b}\ \circledast$
10	KQQTVTLLPAADLDDFSKQLQ	N	388	408		11.0%	52.0%	-	-	-	+	$\circ\ \circ^{d}$
11	LPFNDGVYFASTEKSNIIRGW	S	84	104		58.0%	41.0%	-	+	-	-	$\ast$
12	PLVDLPIGINITRFQTLLALH	S	225	245		65.0%	62.0%	+	-	+	+	$\circledast\ \circledast^{d}$
13	GAAAYYVGYLQPRTFLLKYNE	S	261	281		88.0%	38.0%	+	+	+	-	$\ast^{b}\ \ast^{d}\ \ast^{bd}$
14	LTDEMIAQYTSALLAGTITSG	S	865	885		42.0%	73.0%	+	+	+	+	$\circledast^{d}\ \circledast^{bd}$
15	LSSNFGAISSVLNDILSRLDK	S	966	986		59.0%	62.0%	+	+	-	+	$\circledast^{b}$
16	VTQQLIRAAEIRASANLAATK	S	1008	1028		30.0%	81.0%	-	+	-	+	$\circ\ \circ^{d}\ \circ^{b}\ \circ^{bd}$
17	NYNYLYRLFRKSNLKPFERDI	S	448	468	456-FRKSNLKPFERDISTEIY-473	77.0%	38.0%	+	-	+	-	$\boxed{\ast}^{d}\ \boxed{\ast}^{bd}\ \boxed{\circ}\boxed{\circledast}$
18	YRLFRKSNLKPFERDISTEIY	S	453	473	456-FRKSNLKPFERDISTEIY-473	78.0%	23.0%	+	-	-	-	$\square\ \boxed{\ast}\ \boxed{\ast}^{b}\ \boxed{\circ}$ $\boxed{\circledast}$
19	KPFERDISTEIYQAGSTPCNG	S	462	482	456-FRKSNLKPFERDISTEIY-473	20.0%	21.0%	-	+	-	-	$\boxed{\circ}^{b}$
20	QFGRDIADTTDAVRDPQTLEI	S	564	584	580-QTLE-583	0.0%	0.0%	-	-	-	-	$\square$
21	PQTLEILDITPCSFGGVSVIT	S	579	599	580-QTLE-583	13.0%	0.0%	-	-	-	-	$\boxed{\ast}$
22	QTLEILDITPCSFGGVSVITP	S	580	600	580-QTLE-583	13.0%	21.0%	-	-	-	-	$\boxed{\circ}\boxed{\circledast}$
23	GFNFSQILPDPSKPSKRSFIE	S	799	819	809-PSKP-812	21.0%	23.0%	-	+	-	-	$\square\ \boxed{\circ}\ \boxed{\circ}^{b}\boxed{\circledast}$
24	PSKPSKRSFIEDLLFNKVTLA	S	809	829	809-PSKP-812	66.0%	0.0%	+	-	-	-	$\boxed{\ast}\boxed{\ast}^{b}$

Fig. S10: T cell and B cell vaccine candidates. (A) *21mer vaccine peptide sets selecting for best CD4^{+}, CD8^{+}, CD4^{+}/CD8^{+}, and B cell epitopes with HLA-I, HLA-II, and total population coverage.* ***(B)*** *Unified list of all selected 21mer vaccine peptides. Vaccine peptides containing predicted ligands for murine MHC alleles (H2-b and H2-d haplotypes) are indicated in their respective columns.*
